# FTO up‐regulation induced by MYC suppresses tumour progression in Epstein‒Barr virus‐associated gastric cancer

**DOI:** 10.1002/ctm2.1505

**Published:** 2023-12-11

**Authors:** Yun‐Yun Xu, Ting Li, Ao Shen, Xiao‐Qiong Bao, Jin‐Fei Lin, Li‐Zhen Guo, Qi Meng, Dan‐Yun Ruan, Qi‐Hua Zhang, Zhi‐Xiang Zuo, Zhao‐lei Zeng

**Affiliations:** ^1^ State Key Laboratory of Oncology in South China Sun Yat‐sen University Cancer Center Guangzhou P. R. China; ^2^ Research Unit of Precision Diagnosis and Treatment for Gastrointestinal Cancer Chinese Academy of Medical Sciences Guangzhou P. R. China; ^3^ Department of Gastroenterology and Urology Hunan Cancer Hospital/The Affiliated Cancer Hospital of Xiangya School of Medicine, Central South University Changsha P. R. China; ^4^ Department of Traditional Chinese Medicine Yuebei People's Hospital Shaoguan P. R. China

**Keywords:** EBV‐associated gastric cancer, FOS, FTO, IGF2BP1, IGF2BP2, m6A, metastasis

## Abstract

**Background:**

Epstein‒Barr virus‐associated gastric cancer (EBVaGC) is regarded as a distinct molecular subtype of GC, accounting for approximately 9% of all GC cases. Clinically, EBVaGC patients are found to have a significantly lower frequency of lymph node metastasis and better prognosis than uninfected individuals. RNA N6‐methyladenosine (m6A) modification has an indispensable role in modulating tumour progression in various cancer types. However, its impact on EBVaGC remains unclear.

**Methods:**

Methylated RNA immunoprecipitation sequencing (MeRIP‐seq) and m6A dot blot were conducted to compare the m6A modification levels between EBVaGC and EBV‐negative GC (EBVnGC) cells. Western blot, real‐time quantitative PCR (RT‐qPCR) and immunohistochemistry were applied to explore the underlying mechanism of the reduced m6A modification in EBVaGC. The biological function of fat mass and obesity‐associated protein (FTO) was determined in vivo and in vitro. The target genes of FTO were screened by MeRIP‐seq, RT‐qPCR and Western blot. The m6A binding proteins of target genes were verified by RNA pulldown and RNA immunoprecipitation assays. Chromatin immunoprecipitation and Luciferase report assays were performed to investigate the mechanism how EBV up‐regulated FTO expression.

**Results:**

M6A demethylase FTO was notably increased in EBVaGC, leading to a reduction in m6A modification, and higher FTO expression was associated with better clinical outcomes. Furthermore, FTO depressed EBVaGC cell metastasis and aggressiveness by reducing the expression of target gene AP‐1 transcription factor subunit (FOS). Methylated FOS mRNA was specifically recognized by the m6A ‘reader’ insulin‐like growth factor 2 mRNA binding protein 1/2 (IGF2BP1/2), which enhanced its transcripts stability. Moreover, MYC activated by EBV in EBVaGC elevated FTO expression by binding to a specific region of the FTO promoter.

**Conclusions:**

Mechanistically, our work uncovered a crucial suppressive role of FTO in EBVaGC metastasis and invasiveness via an m6A‐FOS‐IGF2BP1/2‐dependent manner, suggesting a promising biomarker panel for GC metastatic prediction and therapy.

## Graphical abstract

FTO elevation in Epstein‒Barr virus‐associated GC contributes to favourable prognosis in patients.

FTO restrains tumour metastasis and aggressiveness in EBVaGC by down‐regulating FOS in an m6A‐dependent manner.

The m6A readers IGF2BP1/2 bind FOS nascent transcripts and maintain FOS mRNA stability.

Targeting the FTO⊣FOS/ IGF2BP1/2 axis renders a promising strategy for GC therapy.

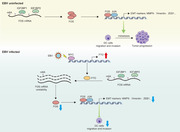

## 1 INTRODUCTION

Gastric cancer (GC) is the fifth most common malignancy globally and the fourth leading cause of cancer death.^1^ Considering the histological heterogeneity of GC, The Cancer Genome Atlas (TCGA) divided it into four subtypes according to a molecular classification in 2014, and one of the distinct molecular subtypes, Epstein‒Barr virus‐associated gastric cancer (EBVaGC), named due to EBV infection in GC cells, comprises approximately 9% of all GC cases.^2^, ^3^, ^4^ EBV, also known as human herpesvirus 4, is widespread among the human population, with over 90% of adults establishing life‐long latent infection. EBVaGC belongs to latency I and II and constitutively expresses latent gene products, such as EBV‐encoded small RNAs (EBERs), EBV nuclear antigen 1 (EBNA1) and latent membrane protein 2A (LMP2A).^5^, ^6^, ^7^, ^8^, ^9^ Furthermore, EBVaGC often shows a molecular profile including PIK3CA mutation, DNA hypermethylation, JAK2, PD‐L1 and PD‐L2 gene amplification, and the clinicopathological features of EBVaGC are male dominance and proximal stomach susceptibility.^10^, ^11^, ^12^ More importantly, compared with EBV‐negative GC (EBVnGC) patients, infected individuals tend to have a significantly lower incidence of lymph node metastasis and favourable clinical outcomes.^13^, ^14^, ^15^, ^16^ Therefore, exploring the underlying mechanism that drives the lower frequency of GC metastasis after EBV infection will provide novel therapeutic targets for GC treatment and may be beneficial for the prognostic prediction of GC patients.

N6‐methyladenosine (m6A) modification has been identified as the most enriched internal transcriptional modification in eukaryotic cells and mainly consists of m6A methyltransferases (also called writers), such as METTL3, METTL14 and WTAP, demethylases (also called erasers), such as FTO and ALKBH5, and m6A binding proteins (also called readers), including YTHDF1/2/3, YTHDC1/2 and IGF2BP1/2/3.^17^, ^18^, ^19^, ^20^, ^21^ Growing evidence indicates that dysregulated m6A modification is associated with tumour proliferation, differentiation, invasion and metastasis in various human cancers.^18^, ^22^, ^23^ In our previous study, methyltransferase‐like 3 (METTL3) facilitated colorectal carcinoma (CRC) progression through increasing SOX2 expression to maintain the CRC stemness phenotype in an m6A‐IGF2BP2‐dependent machinery.^24^ Moreover, other studies found that METTL14 significantly elevated the target gene miRNA126 by positively modulating the pri‐miRNA126 process, thereby playing a tumour‐suppressor role in hepatocellular carcinoma and eventually inhibiting tumour invasion and metastasis.^25^, ^26^ These results highlight the functional importance of the m6A modification regulatory mode in different tumours, which is a promising novel direction of tumour therapy. However, little is currently known regarding the roles and regulatory mechanisms of m6A modification in EBVaGC progression.

Here, we first detected that the m6A demethylase Fat mass and obesity‐associated protein (FTO) was highly expressed in EBVaGC cells, leading to the down‐regulation of m6A modification, and increased FTO expression was correlated with favourable prognosis in EBVaGC patients. Additionally, we demonstrated the suppressive role of FTO in EBVaGC invasiveness and metastasis by repressing the expression of its downstream target gene AP‐1 transcription factor subunit (FOS). Insulin‐like growth factor 2 mRNA binding protein 1/2 (IGF2BP1/2) was found to promote FOS transcript stability and prolong its half‐life. Furthermore, we verified that the transcription factor MYC activated by EBV induced the transcriptional activity and expression of FTO in EBVaGC. Overall, our study revealed the significance of FTO‐FOS‐IGF2BP1/2 as a prospective biomarker panel for GC metastatic prediction and elucidated the regulatory mechanism of the reduced metastasis in EBVaGC from the perspective of epigenetics.

## 2 MATERIALS AND METHODS

### 2.1 Tumour tissues and patient information

Tissue specimens in this work were collected from GC patients undergoing radical surgery at Sun Yat sen University Cancer Center (SYSUCC), which included a total of 331 paraffin‐embedded samples divided into 159 EBVaGC specimens and 160 EBVnGC samples. Of these, fresh frozen tumour tissues were available in 138 cases, including 73 EBVaGC samples for RNA sequencing (RNA‐seq) (62 of which were used for real‐time quantitative polymerase chain reaction (RT‐qPCR) analysis), and 65 EBVnGC tissues for RT‐qPCR analysis. The clinical information of the patients was obtained from medical records and is summarized in Table S1. The EBV infection status of the enrolled patients was determined by in situ hybridization of EBERs. The study was approved by our Institutional Research Ethics Committee (number: GZR2020‐237).

### 2.2 Cell lines and cell culture

The EBVnGC cell line AGS was originally obtained from the American Type Culture Collection (ATCC, Rockville, MD, USA), and the EBVaGC cell lines AGS B95.8 and AGS AKATA were kindly provided by Professor Janet E. Mertz (University of Wisconsin‐Madison, USA). All the cells were maintained in Ham's F12 (C11765500BT, Gibco) medium with 10% foetal bovine serum (085‐150, Wisent) and 1% penicillin/streptomycin (450‐201‐EL, Wisent) at 37°C in a 5% CO2 cell culture incubator. AGS B95.8 cells were cultured with an additional 100 µg/mL of Hygromycin B (HY‐B0490, MCE) and AGS AKATA cells supplemented with 400 µg/mL of G418 (HY‐17561, MCE). All cells were authenticated by short tandem repeat fingerprinting at the Medicine Lab of the Forensic Medicine Department of Sun Yat‐sen University (Guangzhou, China) and were tested negatively for mycoplasma contamination before use.

### 2.3 In vivo tumorigenesis and metastasis models

All female B‐NDG mice (4–5 weeks old) used in this study were purchased from Biocytogen JiangSu Co., Ltd. To evaluate the tumorigenic effect of FTO, FTO knockdown or control AGS B95.8 cells (1 × 10^7^ suspended in 150 µL of PBS) were subcutaneously injected into the flanks of B‐NDG mice. The diameter and width of the tumours were measured every 4 days and used to estimate the tumour volumes by the standard formula: 0.5 × length × width^2^. At the end stage, the tumours were removed, imaged and weighed.

To investigate the peritoneal dissemination ability of EBVaGC cells, intraperitoneal injection was performed. Briefly, 1 × 10^7^ FTO silencing or control EBVaGC cells suspended in 0.4 mL of PBS were injected into the peritoneal cavity of each mouse. Eight weeks later, all the mice were sacrificed, and the abdominal and intestinal metastatic nodules were excised, counted, photographed and paraffin embedded.

For the lung metastasis model, 200 µL of 1 × 10^6^ luciferase‐labelled EBVaGC cells from different groups were directly injected into the tail vein of B‐NDG mice, and distant and lung metastasis were evaluated using bioluminescent imaging. After 6 or 8 weeks, the mice were euthanized, and the lungs were embedded in paraffin and subjected to haematoxylin and eosin (H&E) staining to record the micrometastatic nodules using a microscope. All animal experiments were approved by our Institutional Animal Care.

### 2.4 RNA interference, lentivirus and plasmid transfection

Small interfering RNAs (siRNAs) targeting FTO, FOS, IGF2BP1, IGF2BP2 and MYC were synthesized by RiboBio (Guangzhou, China) and were then transfected using Lipofectamine RNAiMAX (13778150, Invitrogen) following the manufacturer's protocol. The lentiviruses for FTO overexpression and short hairpin RNAs (shRNAs) were purchased from OBiO Technology (Shanghai, China). GC cells were infected by lentiviruses with 5 µg/mL polybrene and selected with 3 µg/mL puromycin (S7417, Selleck) for 1 week. All the sequences of siRNAs and shRNAs are listed in Table S2. Moreover, an N‐terminal Flag‐tagged FTO overexpression wild‐type (WT) plasmid, FTO double mutant (H231A/D233A) plasmid, FOS overexpression plasmid, Flag‐tagged expression vectors for MYC overexpression and luciferase reporter plasmids containing the FTO promoter WT or deletion mutant were constructed by OBiO Technology. The plasmids were transduced into the cells using ViaFect Transfection Reagent (E4982, Promega) according to standard procedures.

### 2.5 m6A dot blot

Total RNA was extracted with TRIzol reagent (15596018, Invitrogen) following the manufacturer' protocol. RNA samples were denatured at 95°C for 3 min and then placed on ice immediately. The samples were loaded onto an Amersham Hybond‐N+ membrane (RPN303B, GE Healthcare) before UV cross‐linking. Whereafter, the membrane was blocked with 5% nonfat milk in PBST buffer for 1 h, while the loading control membrane was stained with .02% methylene blue (HY‐14536, MCE) at room temperature. The experimental membrane was subsequently incubated with m6A antibody overnight at 4°C and visualized by Pierce ECL Plus Western Blotting Substrate (Thermo Fisher Scientific, Waltham, USA) after incubation with HRP‐conjugated goat anti‐rabbit IgG for 1 h at room temperature.

### 2.6 RNA stability assay

EBVaGC cells of different groups were plated onto 12‐well plates overnight and treated with actinomycin D (ActD) (S8964, Selleck) at a final concentration of 10 µg/mL. Cells were then collected at 0, 15, 30 and 45 min after treated by ActD. Total RNA was extracted by TRIzol reagent and analysed using qPCR. The FOS mRNA expression for each group at the indicated time was normalized to β‐Actin. The mRNA half‐life was determined by linear regression analysis, and the percent mRNA remaining was plotted using Prism 8.0 software (GraphPad Software, CA, USA).

### 2.7 Methylated RNA immunoprecipitation qPCR (MeRIP‐qPCR)

The level of m6A modification of transcripts was evaluated by methylated RNA immunoprecipitation (MeRIP)‐qPCR assay. More than 50 µg of total RNA was sheared into approximately 100‐nt‐long fragments by RNA fragmentation reagents (AM8740, Invitrogen), and approximately 1/10 of the RNA was conserved as the input control for further qPCR analysis. Pierce Protein A/G Magnetic Beads (88803, Thermo Scientific) were prewashed, and then, 5 µg of anti‐m6A antibody (202003, Synaptic Systems) or anti‐IgG antibody was added and incubated with gentle rotation at 4°C for 2 h. After washing, the beads were mixed with the RNA fragments in 1× immunoprecipitation buffer on a rotator at 4°C overnight, and proteinase K buffer was used to digest the antibody. The methylated mRNAs were precipitated with 5 mg of glycogen and 3 M sodium acetate in 100% ethanol at −80°C overnight and subsequently reverse transcribed into cDNA. Finally, m6A enrichment in RNA samples was calculated by qPCR analysis and normalized to the input. The primers used are listed in Table S3.

### 2.8 RNA pulldown assay

RNA was first transcribed in vitro using the MEGAscript T7 Transcription Kit (AM1334, Invitrogen) and then end‐labelled with desthiobiotin using the Pierce RNA 3′ End Desthiobiotinylation Kit (20163, Invitrogen). Subsequently, an RNA pulldown assay was conducted using the Pierce Magnetic RNA‐Protein Pull‐Down Kit (20164, Invitrogen) following the suppliers’ instructions. Concisely, up to 50 pmol of biotinylated RNA was mixed with 2 mg of protein lysates and 50 µL of prewashed streptavidin beads. After incubation for 1 h at 4°C and three washes, the streptavidin beads were heated with 50 µL of elution buffer, and the retrieved protein was used for western blotting assays.

### 2.9 m6A motif prediction and m6A mutation assay

The potential m6A modification sites were predicted by analysing the MeRIP sequencing (MeRIP‐seq) data and applying the online tool SRAMP (http://www.cuilab.cn/sramp/). For the m6A mutation assay, full‐length FOS transcripts, the FOS CDS region, the FOS three prime untranslated region (3′‐UTR), and the m6A motif‐deleted CDS and 3′‐UTR regions were cloned into pcDNA3.1 established by OBiO Technology (Shanghai, China) and further used for the RNA pulldown assay. The special sequences are summarized in Table S4.

### 2.10 Chromatin immunoprecipitation assay

Chromatin immunoprecipitation (ChIP) assays were performed using a Magnetic Bead ChIP kit (17‐10085, Merck Millipore) according to the manufacturer's protocol. A total of 1 × 10^7^ cells were collected and cross‐linked using 1% formaldehyde. The lysates were then sonicated to shear DNA to sizes of 200–500 bp, and equal aliquots of isolated chromatin were incubated with 5 µg of anti‐MYC antibody or anti‐IgG antibody. The DNA fragments interacting with MYC or the negative control were isolated for further analysis, and finally, qPCR and nuclear acid electrophoresis assays were carried out to explore the MYC‐binding site on the FTO promoter regions. The primers used are shown in Table S3.

### 2.11 Luciferase reporter assay

The luciferase activities were determined via the Dual‐Luciferase Reporter Assay System (E1910, Promega). In brief, EBVaGC cells were cotransfected with Renilla as an internal control, luciferase reporter plasmids FTO promoter WT or deletion mutant in combination with MYC plasmid or MYC siRNA using Lipofectamine 3000 (L3000015, Invitrogen). The luciferase activities were then detected according to the manufacturer's instructions.

### 2.12 Statistical analysis

All in vitro experiments were performed three times or more, and data are presented as the mean ± standard deviation. A two‐tailed Student's *t* test was used to analyse the differences between two independent groups, while multigroup comparisons were determined by two‐way analysis of variance. For survival analysis, the Kaplan‒Meier method and the log‐rank test were carried out. Pearson's correlation analysis and the chi‐square test were conducted to evaluate the correlations between two variables. The data were analysed using GraphPad Prism 8.0 (GraphPad Software, CA, USA). The indicated *p* values (*p < 0.05, ** *p* < 0.01 and ***p < 0.001) were considered to be statistically significant.

## 3 RESULTS

### 3.1 FTO is up‐regulated in EBVaGC and associated with good prognosis

**FIGURE 1:**
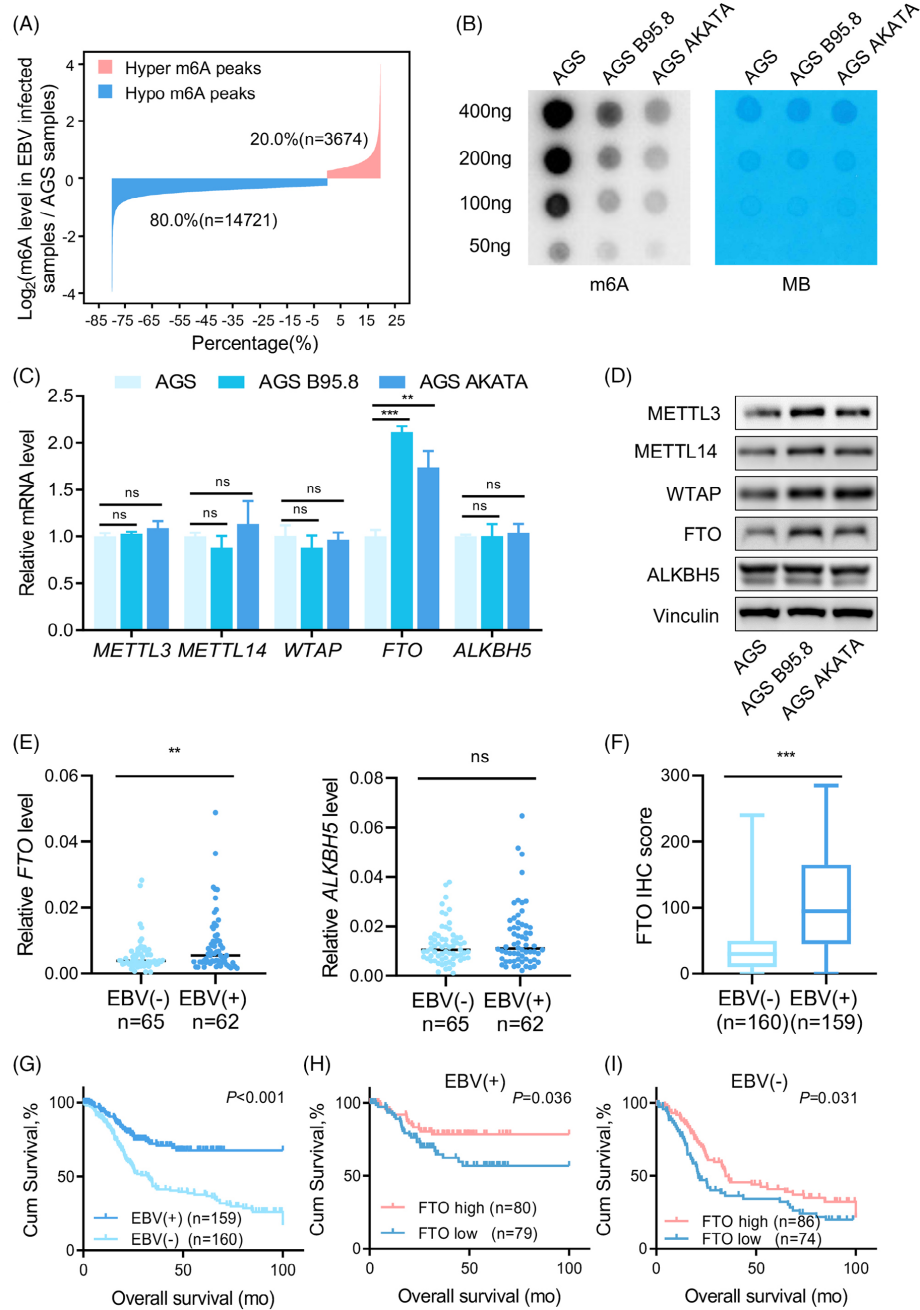
FTO is up‐regulated in EBVaGC and associated with good prognosis. (A) Bar plot showing the distribution of methylation levels of dysregulated m6A peaks in EBVaGC cells (AGS B95.8 and AGS AKATA) compared with EBVnGC cells (AGS) with a fold‐change ≥1.2. (B) RNA m6A dot blot assays using total RNA of EBVaGC and EBVnGC cells. Methylene blue (MB) staining served as a loading control. (C and D) Real‐time PCR analysis (C) and immunoblotting assay (D) of m6A methyltransferase (METTL3, METTL14 and WTAP) and demethylase (FTO and ALKBH5) expressions in EBVaGC cells and EBVnGC cells. (E) Real‐time quantitative PCR (RT‐qPCR) analysis of mRNA expression of FTO and ALKBH5 in EBVaGC (*n* = 62) and EBVnGC tumour samples (*n* = 65) from the Sun Yat‐sen University Cancer Center (SYSUCC) cohort. (F) Immunohistochemistry (IHC) staining scores of FTO expression in EBVaGC (*n* = 159) and EBVnGC tumour tissues (*n* = 160) from the SYSUCC cohort. (G) Kaplan–Meier analysis of overall survival (OS) in GC patients from SYSUCC according to EBV status. (H and I) Kaplan–Meier analysis of OS in EBVaGC patients (H) and EBVnGC patients (I) with different FTO expression levels from the SYSUCC cohort. The FTO level was categorized as ‘high’ and ‘low’ based on the median score (median score = 95) in EBVaGC tumour tissues and the median score (median score = 30) in EBVnGC tumour tissues. The data in (C, E and F) are presented as the means ± SDs. *p*‐Values were determined by Student's *t* test (C, E and F) and calculated by Kaplan–Meier analysis with the log‐rank test (G–I). *p < 0.05; **p < 0.01; ***p < 0.001. Vinculin was used as a loading control.

To detect the changes in RNA m6A modification in EBVaGC cells, we firstly identified the existence of virus in the EBVnGC cell line AGS and two EBVaGC cell lines, AGS B95.8 and AGS AKATA. The EBV‐specific genes including EBNA1 and LMP2A were expressed in EBV‐positive but not in EBV‐negative GC cells (Figure S1A). MeRIP‐seq and RNA‐seq between EBVaGC and EBVnGC cells were then performed, and the analysis showed that there were 14,721 (80.0%) hypomethylated m6A peaks and 3,674 (20.0%) hypermethylated m6A peaks in AGS B95.8 and AGS AKATA cells compared to AGS cells (Figure 1A), which indicated that the m6A peaks were generally down‐regulated in GC cells with EBV infection. We further evaluated the actual m6A modification between EBVaGC and EBVnGC using a m6A dot blot assay. Consistent with the sequencing results, m6A modification was significantly reduced in AGS B95.8 and AGS AKATA cells compared with AGS cells (Figure 1B), and the decreased m6A abundance was also verified in the EBVaGC specimens compared to the EBVnGC specimens (Figure S1B), possibly due to the decrease of m6A writers or the increase of erasers in EBVaGC.

To explore the underlying mechanism of m6A modification reduction in EBVaGC, we investigated the expression of m6A methyltransferases (METTL3, METTL14 and WTAP) and demethylases (FTO and ALKBH5) between EBVaGC and EBVnGC cells. The qPCR results indicated that only FTO transcripts were increased in EBVaGC cells, but not for other m6A writers or erasers (Figure 1C). Moreover, the protein levels of m6A methyltransferases and FTO were higher in EBVaGC cells than in EBVnGC cells (Figure 1D). Further validation showed that FTO was elevated in EBVaGC tumour tissues from the SYSUCC cohort, whereas the other demethylase ALKBH5 and m6A methyltransferases all remained unchanged (Figure 1E and Figure S1C) (clinicopathological information is summarized in Table S1). These data suggested that high expression of FTO may lead to a reduction of m6A modification in EBVaGC.

To identify the clinical implication of FTO in GC, we selected archived EBVaGC and EBVnGC tissue sections from SYSUCC for FTO IHC staining (Figure S1D). As expected, the overall FTO staining score of EBVaGC tissues was higher than that of EBVnGC tissues (Figure 1F). Moreover, EBVaGC patients had longer overall survival than EBVnGC patients, and the prognosis of EBVaGC or EBVnGC patients with higher FTO expression was also better (Figure 1G–I). The above results suggested that the m6A demethylase FTO was significantly elevated in EBVaGC and might be a potential prognostic indicator for GC patients.

### 3.2 FTO restrains EBVaGC cell migration and invasion in vitro

**FIGURE 2:**
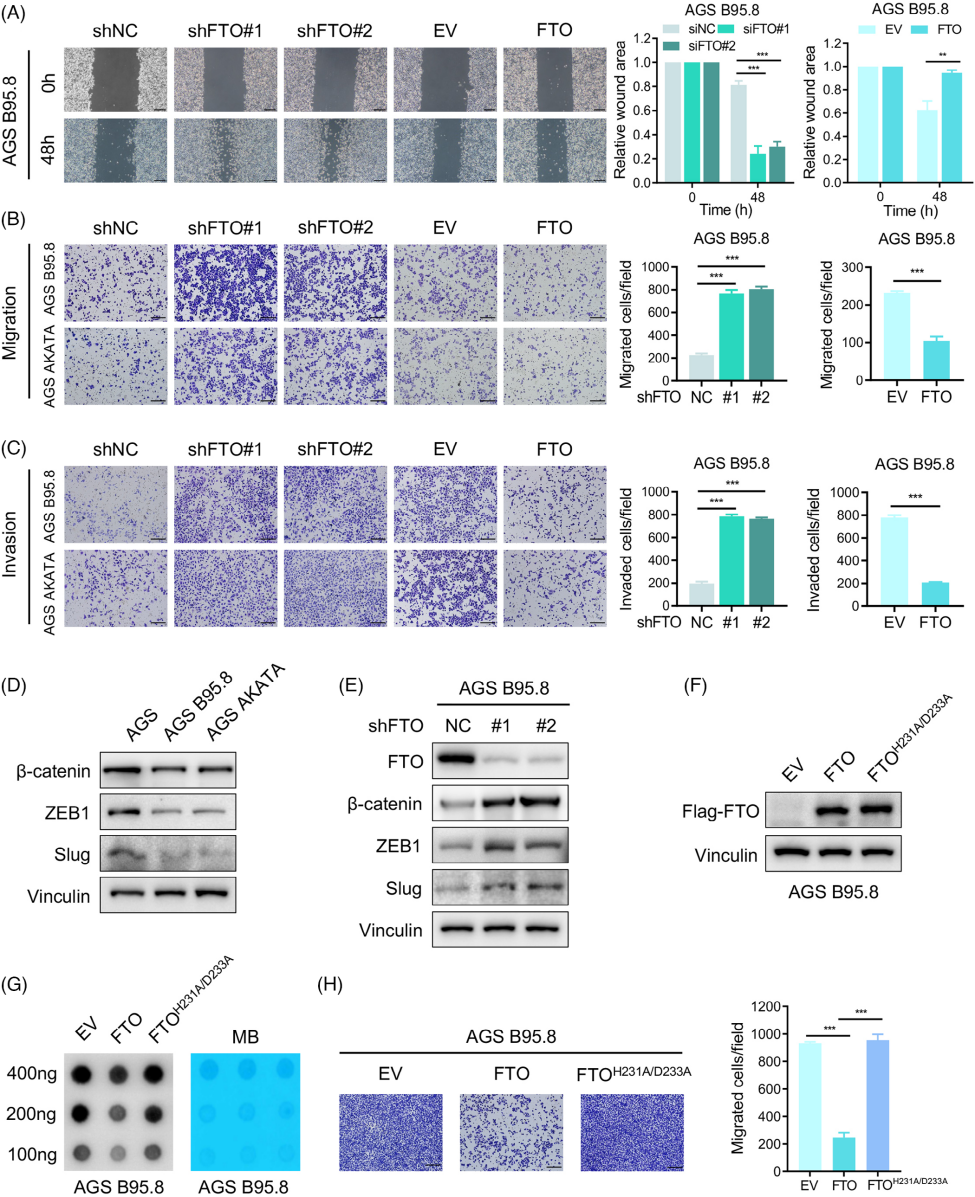
FTO restrains EBVaGC cell migration and invasion in vitro. (A) Wound healing assays of AGS B95.8 cells with FTO silencing and FTO overexpression were recorded and quantitatively analysed. Scale bar: 200 µm. (B and C) Images and quantification of cell migration (top) and invasion (bottom) assays of FTO‐knockdown and FTO‐overexpressing EBVaGC cells. Scale bar: 200 µm. (D) Western blotting analysis was used to detect EMT markers (β‐Catenin, ZEB1, Slug) in EBVaGC and EBVnGC cells. (E) The protein levels of EMT markers (β‐Catenin, ZEB1, Slug) in AGS B95.8 cells after FTO deficiency were measured using immunoblotting. (F) Western blotting assay of FTO in AGS B95.8 cells overexpressing wild‐type and catalytic mutant FTO. (G) RNA m6A dot blot assay of wild‐type and catalytic mutant FTO‐overexpressing AGS B95.8 cells. MB staining served as a loading control. (H) The migration ability of AGS B95.8 cells overexpressing wild‐type and catalytic mutant FTO was determined (left), and the cell migration assay results were quantitatively analysed (right). Scale bar: 200 µm. The data in (A–C and H) are presented as the means ± SDs. *p*‐Values were determined by Student's *t* test. *p < 0.05; **p < 0.01; ***p < 0.001. Vinculin was included as a loading control.

The expression discrepancy of FTO prompted us to investigate its function and role in the progression of EBVaGC. We firstly constructed stable FTO‐overexpressing and FTO‐knockdown AGS B95.8 and AGS AKATA cell lines (Figure S2A,B). A series of experiments were conducted to verify the effect of FTO on migration, invasion and proliferation in EBVaGC. The metastatic ability of EBVaGC cells was obviously promoted after FTO deficiency; conversely, ectopic expression of FTO impaired cell migration in EBVaGC cells, as shown by wound healing and transwell migration assays (Figure 2A,B and Figure S2C,D). Similar results were also observed in the cell invasion assay (Figure 2C and Figure S2E). Moreover, we detected that EBV infection in GC cells significantly decreased the expressions of β‐catenin, ZEB1 and Slug (Figure 2D), all of which are epithelial‐mesenchymal transition (EMT)‐related markers. In addition, we found that FTO silencing in EBVaGC cells effectively enhanced the protein levels of these EMT‐related markers (Figure 2E and Figure S2F), and FTO deletion successfully promoted EMT through inducing the disruption of cell–cell junctions and spindle‐like appearance EBVaGC cells (Figure S2G), which implied that FTO was indispensable for repressing the metastatic potential of EBVaGC cells. However, intriguingly, the MTT assay showed that FTO had no influence on the growth of EBVaGC cells (Figure S2H).

We presumed that FTO suppressed EBVaGC cell metastasis in an m6A‐dependent pattern. To verify our suppose, a double mutant FTO plasmid named FTO^H231A/D233A^ was constructed (Figure 2F and Figure S2I), which completely aborted m6A demethylation activity according to previous reports.^27^, ^28^, ^29^ Correspondingly, m6A levels were dramatically increased in EBVaGC cells with the catalytic mutant FTO relative to wild‐type cells (Figure 2G), and the cell migration assay showed that the m6A demethylation activity of FTO is indispensable for its role in inhibiting migrated ability of EBVaGC cells (Figure 2H and Figure S2J). Together, these results revealed that FTO overexpression dampened EBVaGC cell metastatic capacity via m6A mechanisms in vitro.

### 3.3 FTO suppressed EBVaGC cell metastasis in vivo

**FIGURE 3:**
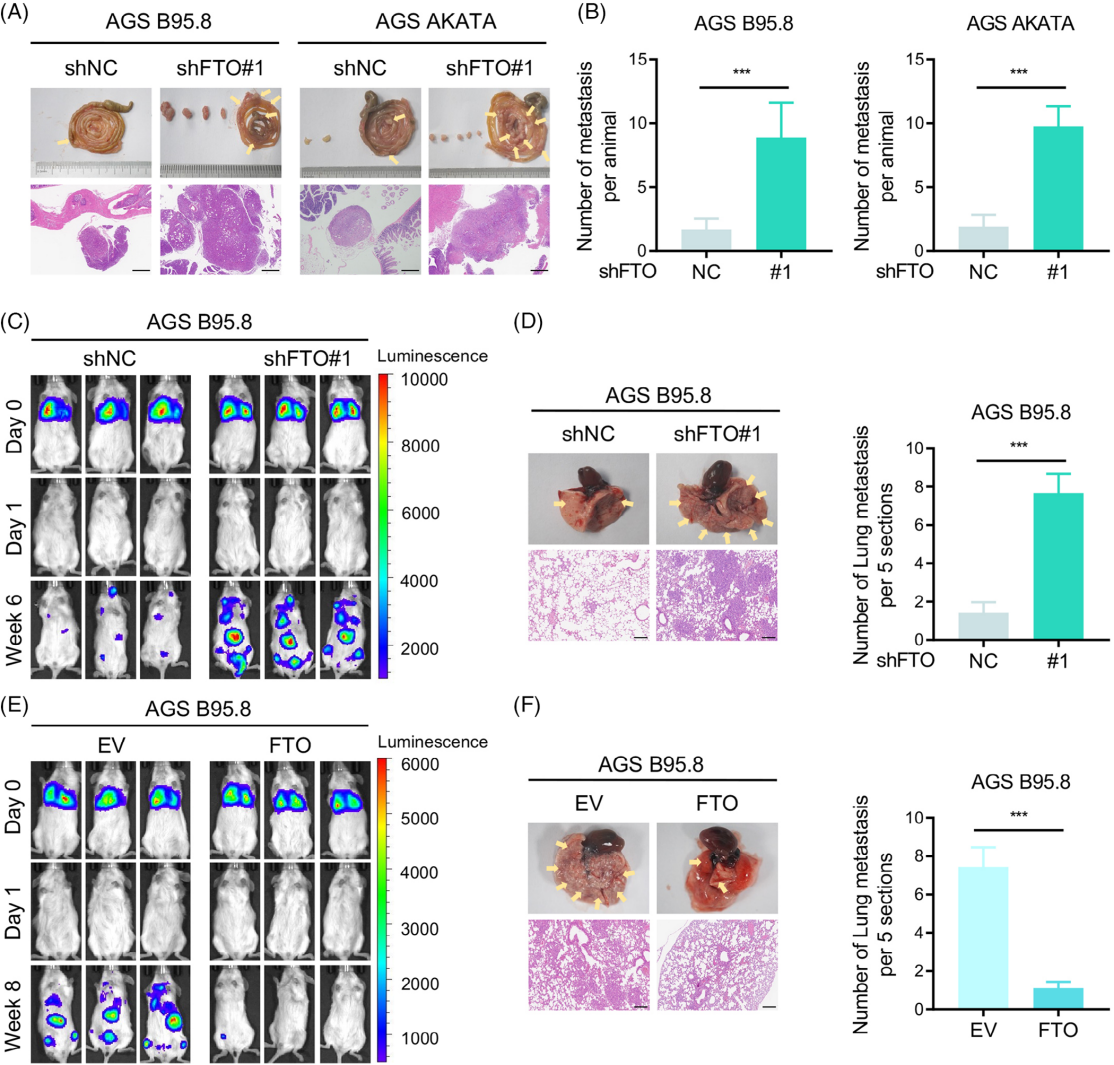
FTO suppressed EBVaGC cell metastasis in vivo. (A) Abdominal and intestinal metastatic nodules of the FTO knockdown and control groups (*n* = 9 per group) were photographed (top), and haematoxylin and eosin (H&E) staining was performed (bottom). Scale bar: 500 µm. (B) Metastatic numbers of AGS B95.8 (left) and AGS AKATA (right) cells were recorded. (C) Bioluminescence imaging of the B‐NDG mouse lung metastasis model with shFTO#1 and shNC luciferase‐labelled AGS B95.8 cells at day 0, day 1 and week 6 (*n* = 9 per group). (D) Representative specimen and H&E staining photographs (left) of lung metastatic nodules from B‐NDG mice injected with FTO‐knockdown and control AGS B95.8 cells via the tail vein, and lung metastatic nodules under a microscope were recorded (right). Scale bar: 200 µm. (E) Representative luciferase imaging of the B‐NDG mouse lung metastatic model in the FTO‐overexpressing (FTO) and empty vector (EV) groups at day 0, day 1 and week 8 (*n* = 9 per group). (F) Representative specimen and H&E staining photographs (left) of the metastatic nodules in the lung, and lung metastatic nodules were recorded (right). Scale bar: 200 µm. The data in (B, D and F) are presented as the means ± SDs. *p*‐Values were determined by Student's *t* test. *p < 0.05; **p < 0.01; ***p < 0.001.

To assess the effect of FTO levels on tumour metastasis in vivo, we conducted two metastasis models, an abdominal transferred carcinoma model and a lung metastasis model. The data showed that FTO knockdown obviously promoted tumour cell metastasis in the abdominal and intestinal walls after peritoneal injection of EBVaGC cells (Figure 3A,B). Moreover, luciferase‐labelled EBVaGC cells overexpressing and silencing FTO were injected through the tail vein of B‐NDG mice. After 6 weeks compared with the control group, FTO deficiency remarkably enhanced EBVaGC cell distant metastasis, as shown by bioluminescence imaging, and increased the number and size of lung metastatic nodules (Figure 3C,D and Figure S3A,B). Oppositely, FTO overexpression in AGS B95.8 cells inhibited distant metastasis, and these cells formed few lung metastatic lesions (Figure 3E,F). Considering that FTO showed no effect on tumour cell proliferation in vitro (Figure S2G), we applied a B‐NDG mouse subcutaneous xenograft model to verify its function in vivo and found that there was no significant difference in the tumour growth rate and tumour weight after FTO down‐regulation in AGS B95.8 cells (Figure S3C–E). Thus, our results indicated that FTO is critical for suppressing tumour metastatic potential in vivo.

### 3.4 FOS is a functionally critical downstream target of FTO in EBVaGC metastasis

**FIGURE 4:**
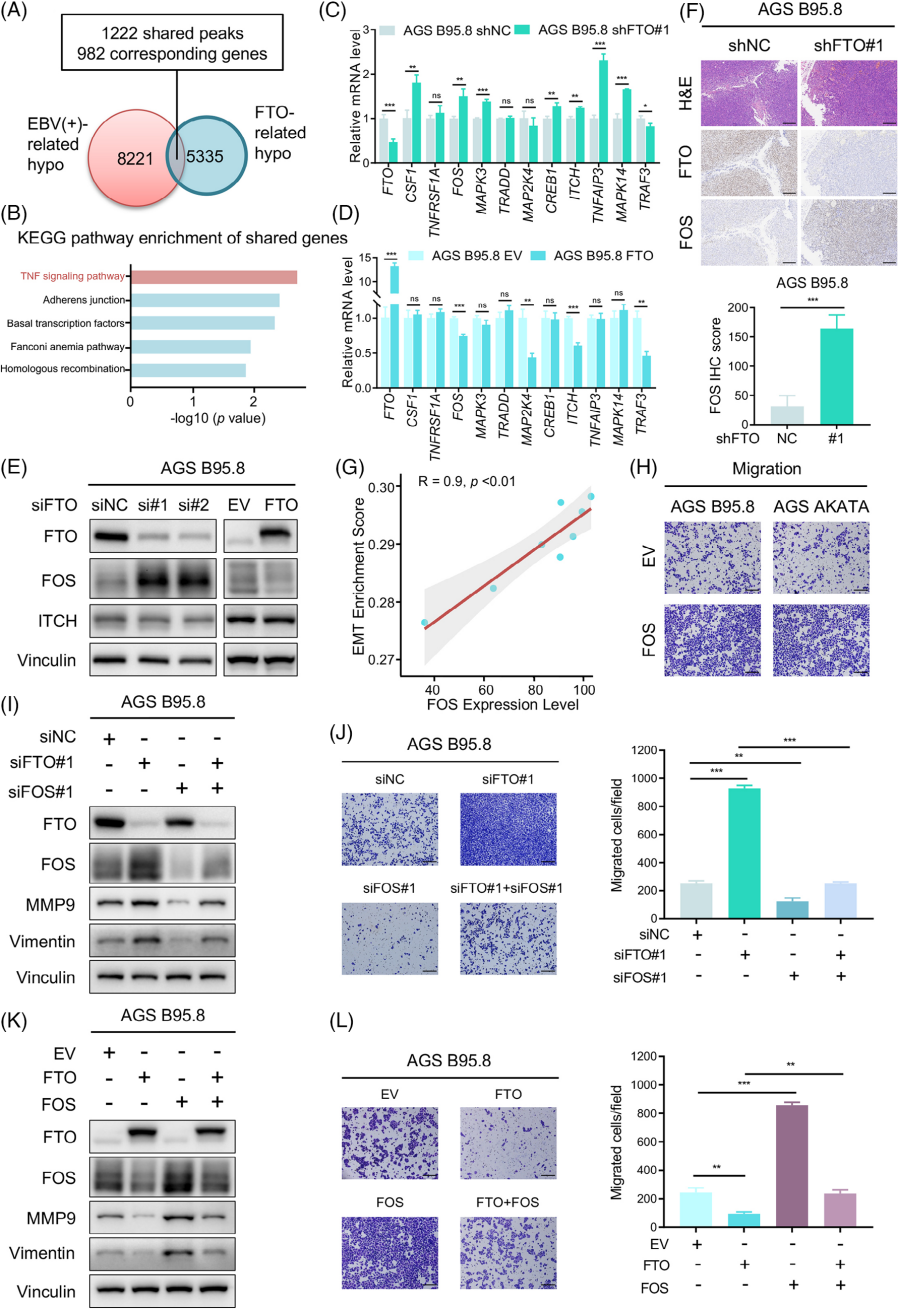
FOS is a functionally critical downstream target of FTO in EBVaGC metastasis. (A) Venn diagram illustrating the shared peaks between EBV‐related hypo peaks and FTO‐related hypo peaks. A total of 1222 shared peaks corresponding to 982 specific genes were obtained. (B) Kyoto Encyclopedia of Genes and Genomes (KEGG) pathway analysis of the above 982 specific genes, and the enriched top five enriched pathways were the TNF signalling pathway, Adherens junction, Basal transcription factors, Fanconi anaemia pathway and Homologous recombination. (C and D) The  RT‐qPCR assay was performed to assess mRNA expression of the shared genes in the TNF signalling pathway (CSF1, TNFRSF1A, FOS, MAPK3, TRADD, MAP2K4, CREB1, ITCH, TNFAIP3, MAPK14 and TRAF3) with FTO knockdown (C) and FTO overexpression (D) in AGS B95.8 cells. (E) Western blotting analysis of FOS and ITCH protein levels in FTO‐knockdown (left) and FTO‐overexpressing (right) AGS B95.8 cells. (F) Representative H&E and IHC images of FTO and FOS expression in FTO‐deficient and control AGS B95.8 cell‐induced xenograft tumours (top). Scale bar: 200 µm. Quantification of FOS expression (bottom) in the FTO knockdown and control groups (*n* = 9 per group). (G) Scatterplot showing the correlation between FOS expression level and the enrichment scores of EMT pathway obtained from single sample gene set enrichment analysis (ssGSEA) using the RNA‐seq data of AGS, AGS B95.8, FTO‐overexpressing AGS B95.8 and control cells. (H) Images of the cell migration assay of FOS‐overexpressing (FOS) versus empty vector (EV) AGS B95.8 (left) and AGS AKATA (right) cells. Scale bar: 200 µm. (I) Western blotting assay of FTO, FOS, MMP9 and Vimentin protein levels in AGS B95.8 cells transfected with siNC, siFTO#1, siFOS#1 and siFTO#1 + siFOS#1. (J) Representative images (left) and quantification (right) of the transwell migration assay of AGS B95.8 cells with siNC, siFTO#1, siFOS#1 and siFTO#1 + siFOS#1. (K) Immunoblotting assays were conducted to detect FTO, FOS, MMP9 and Vimentin expression in AGS B95.8 cells with EV, FTO, FOS and FTO + FOS. (L) Images (left) and quantification (right) of the cell migration assay of AGS B95.8 cells with EV, FTO, FOS and FTO + FOS. Scale bar: 200 µm. The data in (C, D, F, J and L) are presented as the means ± SDs. *p*‐Values were determined by Student's t test. *p < 0.05; **p < 0.01; ***p < 0.001. Vinculin was included as a loading control.

To investigate the mechanism by which FTO regulates the progression of EBVaGC, we next performed MeRIP‐seq and RNA‐seq in AGS B95.8 cells with or without FTO overexpression. Based on the whole sequencing results, there were 8221 hypomethylated m6A peaks in EBVaGC cells compared to EBVnGC cells, named EBV‐related hypo peaks. Similarly, the data showed that 5335 hypomethylated m6A peaks were observed in FTO‐overexpressing AGS B95.8 cells versus control cells and were called FTO‐related hypo peaks. Overlapping the peaks in these two groups, we noted that 1222 specific peaks, corresponding to 982 genes, were obtained (Figure 4A). We then discovered that the shared genes were most enriched in the tumour necrosis factor (TNF) signalling pathway, in which these genes included CSF1, TNFRSF1A, FOS, MAPK3, TRADD, MAP2K4, CREB1, ITCH, TNFAIP3, MAPK14 and TRAF3, according to Kyoto Encyclopedia of Genes and Genomes pathway analysis (Figure 4B). Subsequently, an RT‒qPCR analysis showed that the candidate genes FOS and ITCH were both regulated by FTO at the transcriptional level (Figure 4C,D and Figure S4A), and further verification showed that only the FOS protein level was negatively correlated with FTO expression using western blotting analysis (Figure S4B and Figure 4E). Moreover, an IHC staining assay was conducted to explore the correlation between FTO and FOS in vivo and revealed that silencing FTO elevated FOS expression in tumour tissues (Figure 4F).

Known as the AP‐1 transcription factor subunit, FOS encodes leucine zipper proteins that dimerise with Jun family proteins, thus forming the AP‐1 transcription factor complex. Consequently, FOS is considered to be involved in cell proliferation, migration, invasion, differentiation, apoptosis and transformation.^30^, ^31^, ^32^, ^33^ Previous studies have reported that FOS participates in tumour metastasis by upregulating the expression of EMT‐related markers as a transcription factor.^34^, ^35^, ^36^, ^37^, ^38^, ^39^ Subsequently, we analysed the RNA‐seq data of the indicated cells through single‐sample gene set enrichment analysis (ssGSEA) and found that FOS expression exhibited a positive correlation with EMT enrichment scores (Figure 4G). Therefore, we speculated that FTO suppressed EBVaGC metastasis by impairing the expression of the target gene FOS. To test this hypothesis, FOS was overexpressed in EBVaGC cells and was detected to apparently expedite cell migration (Figure S4C,D and Figure 4H). Furthermore, we established simultaneous FTO and FOS knockdown or overexpression in EBVaGC cells. MMP9 and Vimentin, known as EMT‐related markers, have been reported to be FOS downstream target genes.^34^, ^40^ Immunoblotting and cell migration assays showed that FOS deletion dramatically reduced the protein levels of MMP9 and Vimentin and dampened EBVaGC cell migration, and FOS knockdown reversed the enhanced effect of cell migration due to FTO deficiency (Figure 4I,J and Figure S4E,F). Similarly, the ectopic expression of FOS in FTO‐overexpressing EBVaGC cells notably elevated these EMT biomarkers expression and accelerated cell metastasis (Figure 4K,L and Figure S4G,H). In summary, these data indicated that FOS was the downstream target gene of FTO, and FTO restrained EBVaGC metastasis through the downregulation of FOS expression.

### 3.5 FOS is regulated by FTO‐dependent m6A demethylation

**FIGURE 5:**
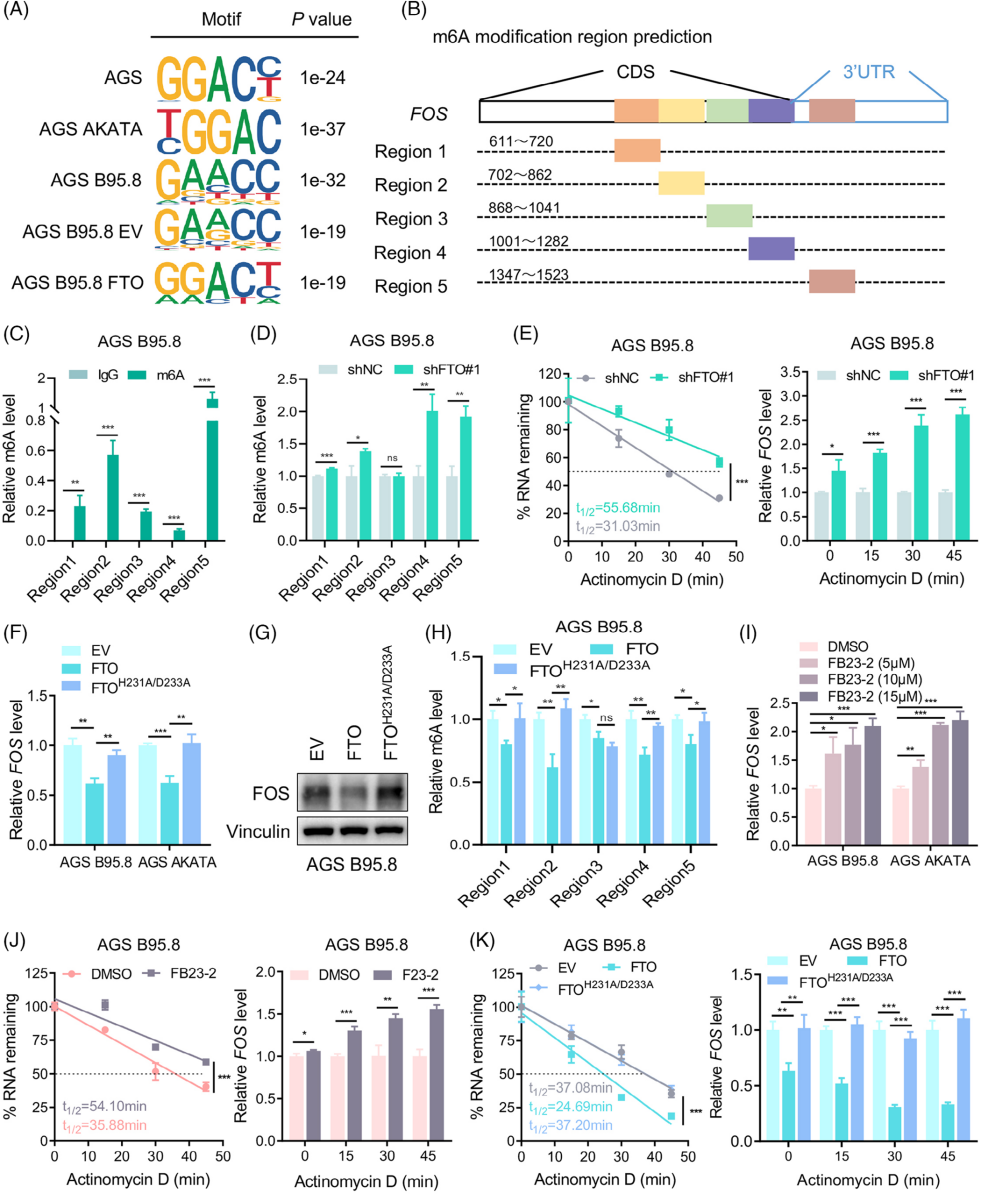
FOS is regulated by FTO‐dependent m6A demethylation. (A) MeRIP‐seq analysis showing canonical RRACH m6A motifs of FOS transcripts in EBVaGC cells and EBVnGC cells. (B) The predicted m6A regions in FOS mRNA. (C) MeRIP‐qPCR analysis of m6A enrichment in regions 1−5 of FOS transcripts in AGS B95.8 cells. (D) MeRIP‐qPCR analysis of m6A enrichment in regions 1−5 of FOS mRNA in FTO‐knockdown and control AGS B95.8 cells. (E) The decay rate (left) and qPCR detection (right) of FOS mRNA at the indicated time point after actinomycin D (ActD) treatment in FTO‐knockdown versus control groups. (F and G)  RT‐qPCR analysis (left) and immunoblotting (right) of FOS expression in EBVaGC cells with empty vector (EV), wild‐type FTO overexpression (FTO) and H231A/D233A‐mutant FTO overexpression (FTO^H231A/D233A^). (H) MeRIP‐qPCR analysis was conducted to detect the relative m6A level of FOS mRNA regions in AGS B95.8 cells transfected with vectors expressing wild‐type or catalytic‐mutant FTO. (I) Relative FOS mRNA expression in EBVaGC cells treated with DMSO, FB23‐2 (5 µM), FB23‐2 (10 µM) and FB23‐2 (15 µM). (J) The decay rate (left) and qPCR assay (right) of FOS mRNA at the indicated times after ActD treatment in FB23‐2 (15 µM) versus DMSO‐treated AGS B95.8 cells. (K) The decay rate of FOS transcripts in AGS B95.8 cells transfected with EV, FTO and FTO^H231A/D233A^ plasmids was analysed by nonlinear regression (left), and the relative FOS mRNA expression of different groups was detected by qPCR analysis at each time point (right). The data in (C–F and H–K) are presented as the means ± SDs. *p*‐Values were determined by Student's *t* test (C, D, F, H, I and relative *FOS* level in E, J and K) and two‐way ANOVA (%RNA remaining in E, J and K). *p < 0.05; **p < 0.01; ***p < 0.001. IgG was used as the negative control and the relative m6A level was normalized by the input in (C, D and H). Vinculin was included as a loading control.

M6A often occurs in an RRACH (R = G or A, H = A, C or U) conserved sequence. The MeRIP‐seq results indicated that canonical RRACH m6A motifs were present in FOS transcripts in EBVaGC and EBVnGC cells (Figure 5A). The above results have shown that FTO delays EBVaGC cell metastatic progression relying on m6A modification, and FOS was identified as an essential substrate of FTO affecting metastasis. To explore the potential mechanism by which FTO regulates FOS, we firstly predicted the m6A modification sites on FOS mRNA according to the MeRIP‐seq data and SRAMP (http://www.cuilab.cn/sramp/) and divided them into five regions, labelled region 1 to region 5, due to their close position (Figure 5B). As shown by MeRIP‐qPCR and RNA stability assays, the m6A abundances of FOS were increased, and the FOS mRNA lifespan was prolonged in GC cells without EBV infection (Figure S5A,B). We next verified the m6A modification of FOS mRNA using a MeRIP‐qPCR assay in EBVaGC cells. The data demonstrated that these predicted regions of FOS mRNA were credibly regions modified with m6A modification (Figure 5C and Figure S5C); meanwhile, the m6A levels of regions 1, 2, 4 and 5 were obviously increased in EBVaGC cells with FTO depletion (Figure 5D and Figure S5D). We then speculated that the m6A‐regulation axis enhances FOS transcript expression through maintaining mRNA stability and reducing its degradation. Consistent with our hypothesis, FOS mRNA expression was gradually increased, and FOS mRNA lifespan was consistently prolonged upon FTO deletion in EBVaGC cells (Figure 5E and Figure S5E), which suggested that FTO inhibited FOS mRNA expression by damaging the mRNA stability of FOS.

To further confirm the function of FTO by m6A demethylase activity, we assessed the mRNA expression and protein level of FOS in EBVaGC cells transfected with wild‐type and H231A/D233A‐mutant FTO plasmids. The data illustrated that FOS protein and transcript expressions were merely downregulated in FTO wild‐type cells (Figure 5F,G and Figure S5F). In parallel, MeRIP‐qPCR validation showed that the m6A level of FOS was visibly elevated in EBVaGC cells with FTO‐mutated plasmids relative to the FTO wild‐type group (Figure 5H). Furthermore, FB23‐2 has been previously reported to be a potent small‐molecule inhibitor against FTO, selectively suppressing its m6A demethylase activity.^41^, ^42^, ^43^ After EBVaGC cells were treated with 5, 10 and 15 µM FB23‐2 for 24 h, FTO mRNA expression was naturally decreased, and FOS transcript levels were markedly increased (Figure S5G and Figure 5I). In addition, we observed that the FOS mRNA decay rate was slower with FB23‐2 treatment (Figure 5J), and the accelerated FOS mRNA decay rate was detected in the FTO wild‐type group but not in the FTO^H231A/D233A^ mutant group compared with the normal control group (Figure 5K). Taken together, these results indicated that FTO inhibited FOS expression by impairing its mRNA stability and shortening its half‐life in an m6A‐dependent manner.

### 3.6 IGF2BP1/2 promotes FOS transcript stability via binding its m6A‐modified mRNA

**FIGURE 6:**
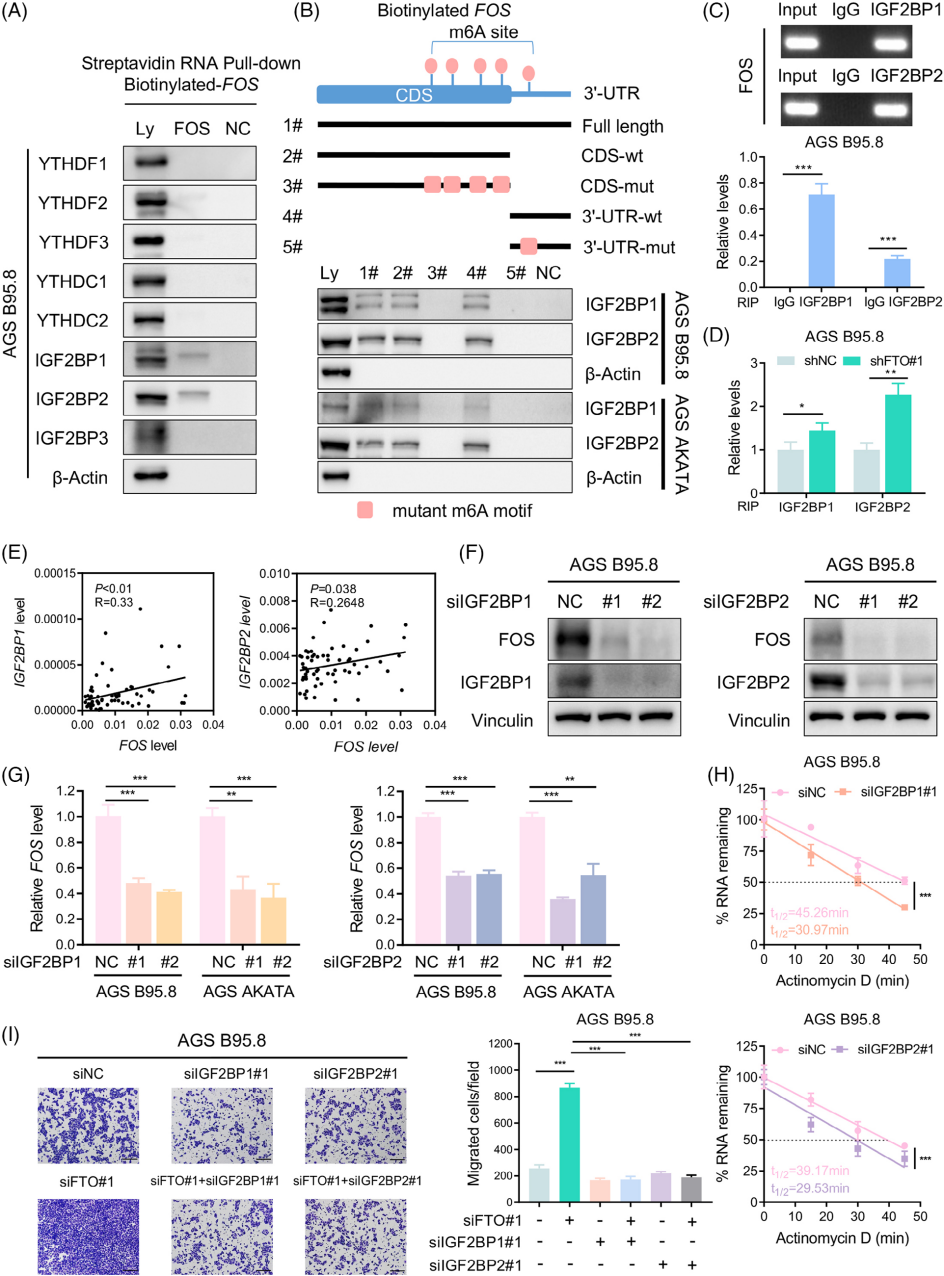
IGF2BP1/2 promotes FOS transcript stability by binding its m6A‐modified mRNA. (A) Western blotting of the YTH family and IGF2BP family with cell lysate (Ly), biotinylated full‐length FOS (FOS) and beads only (NC) in AGS B95.8 cells using an RNA pulldown assay. (B) Western blotting assay of IGF2BP1/2 with cell lysate (Ly), full‐length FOS (#1), the FOS CDS region with or without m6A motif mutation (#2, #3), the FOS 3′‐UTR with or without m6A motif mutation (#4, #5) and beads only (NC) in EBVaGC cells. (C) Agarose gel electrophoresis (top) and qPCR detection (bottom) of RIP assays indicated direct binding between IGF2BP1/2 protein and FOS transcripts in AGS B95.8 cells. (D) The enrichment of IGF2BP1/2 protein binding FOS mRNA in AGS B95.8 cells upon FTO silencing was determined by RIP assays. (E) Q‐PCR assay illustrating that FOS expression was positively correlated with IGF2BP1 (left) and IGF2BP2 (right) expression in 62 EBVaGC specimens from the SYSUCC cohort. (F) Immunoblotting assay of FOS protein levels after IGF2BP1 (left) and IGF2BP2 (right) inhibition in AGS B95.8 cells. (G)  RT‐qPCR analysis of FOS mRNA expression in EBVaGC cells with IGF2BP1 (left) and IGF2BP2 (right) inhibition. (H) The decay rate of FOS transcripts in AGS B95.8 cells after IGF2BP1 knockdown (top) and IGF2BP2 knockdown (bottom). (I) Representative images (left) and quantification (right) of migrated AGS B95.8 cells with siNC, siFTO#1, siIGF2BP1#1, siFTO#1 + siIGF2BP1, siIGF2BP2#1, siFTO#1 + siIGF2BP2#1. Scale bar: 200 µm. The data in (C, D and G–I) are presented as the means ± SDs. *p*‐Values were determined by Student's t test (C, D, G, I), two‐way ANOVA (H), Pearson's correlation analysis and chi‐square test (E). *p < 0.05; **p < 0.01; ***p < 0.001. β‐Actin (A and B) and Vinculin (F) were included as loading controls.

The function of m6A modification in regulating downstream gene expression is conducted mainly by m6A ‘readers’, such as the YT521‐B homology (YTH) domain‐containing family (YTHDF1/2/3, YTHDC1/2) and the insulin‐like growth factor 2 mRNA binding protein (IGF2BP) family (IGF2BP1/2/3).^44^, ^45^, ^46^, ^47^ We used an RNA pulldown assay to identify the FOS‐related m6A readers, and the data showed that IGF2BP1 and IGF2BP2, but not other readers, could specifically bind the FOS full‐length transcripts in AGS B95.8 cells (Figure 6A). Subsequently, the direct binding of IGF2BP1/2 and FOS transcripts was confirmed again in EBVaGC cells (Figure S6A), and we found that the binding was remarkably dampened once the m6A motif was absent (Figure 6B and Figure S6B). Moreover, the RIP assay also demonstrated the interaction between the IGF2BP1/2 protein and FOS mRNA in EBVaGC cells (Figure 6C and Figure S6C), and the binding of IGF2BP1/2 and FOS was significantly strengthened after FTO deficiency (Figure 6D and Figure S6D). In 62 EBVaGC tumour tissues from the SYSUCC cohort, FOS expression was detected to be positively correlated with IGF2BP1/2 expression (Figure 6E), suggesting an underlying positive regulatory mechanism. As expected, the depletion of IGF2BP1/2 could dramatically reduce the protein and mRNA expression of FOS in EBVaGC cells (Figure 6F,G and Figure S6E,F), and the FOS mRNA decay rate was consistently accelerated after IGF2BP1/2 knockdown (Figure 6H and Figure S6G). In addition, a cell migration assay verified that FTO silencing promoted EBVaGC cell metastasis, while these enhanced effects were obviously suppressed upon simultaneous IGF2BP1/2 reduction (Figure 6I). These findings indicated that methylated FOS mRNA was specifically recognized by the m6A “readers” IGF2BP1/2, which maintained FOS expression and enhanced the metastatic capacity of EBVaGC cells via IGF2BP1/2‐dependent FOS transcript stability.

### 3.7 EBV induces FTO expression by the transcription factor MYC in GC

**FIGURE 7:**
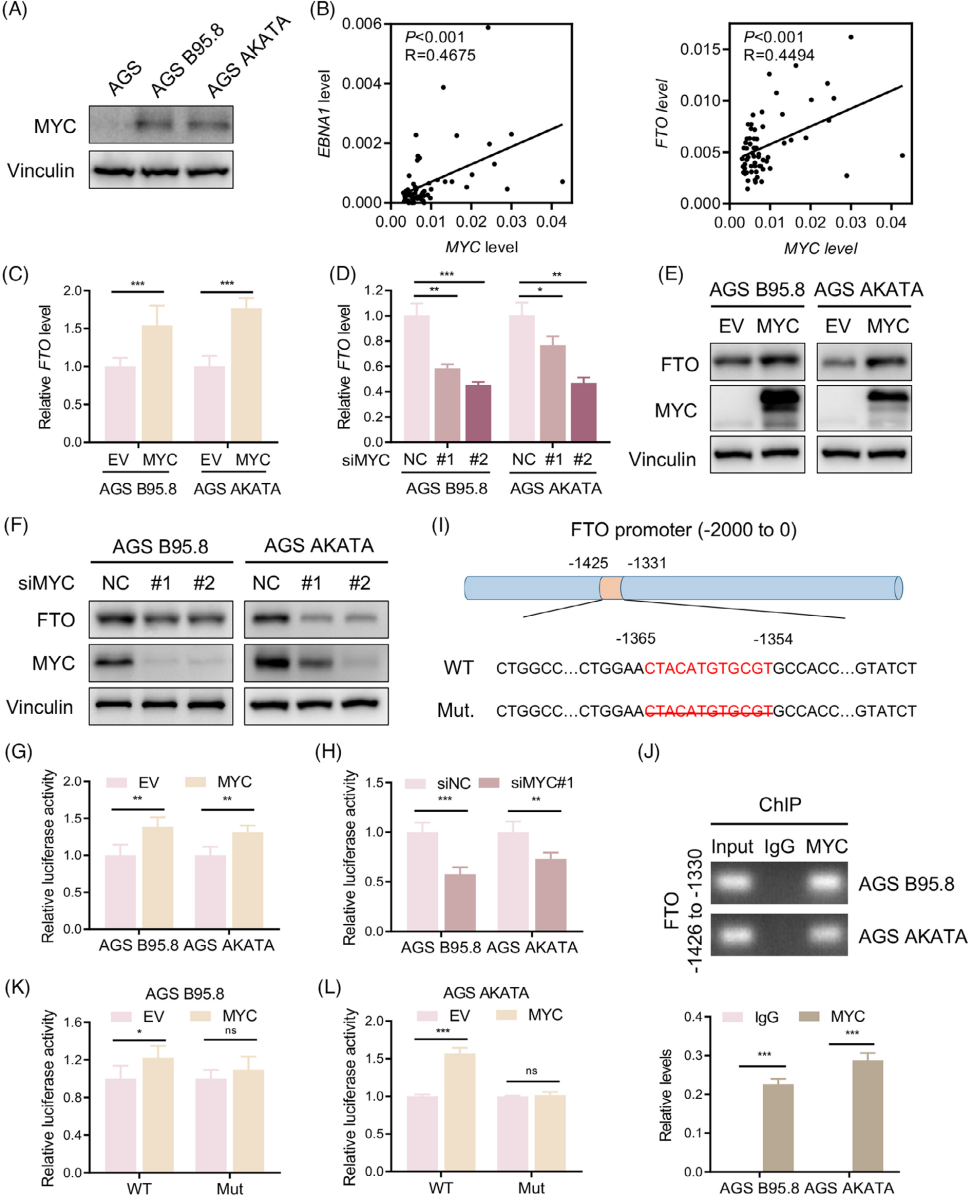
EBV induces FTO expression by the transcription factor MYC in GC. (A) Immunoblotting of MYC protein levels in EBVaGC cells and EBVnGC cells. (B) Correlation of EBNA1 (left) and FTO (right) with MYC mRNA expression in 62 EBVaGC samples from SYSUCC. (C and D)  RT‐qPCR analysis of FTO mRNA levels in EBVaGC cells upon MYC overexpression (C) and MYC knockdown (D). (E and F) Western blotting was performed to assess FTO and MYC protein expression levels in EBVaGC cells with MYC up‐regulation (E) and MYC down‐regulation (F). (G and H) Luciferase reporter assay of FTO transcriptional activity in MYC‐overexpressing (G) and MYC‐knockdown (H) EBVaGC cells. (I) Schematic illustration showing the FTO promoter containing the predicted MYC‐binding sites (−1365 to −1354). The strategy for mutating the FTO promoter is to delete the binding sequences. (J) Agarose gel electrophoresis (top) and qPCR analysis (bottom) of the ChIP assay indicated the enrichment of MYC on the FTO promoter at the predicted region of −1365 to −1354 in EBVaGC cells. (K and L) Luciferase reporter assay showing the transcriptional activity of FTO in AGS B95.8 (K) and AGS AKATA (L) cells overexpressing the FTO wild type and truncation mutant. The data in (C, D, G, H, K and L) are presented as the means ± SDs. *p*‐Values were determined by Student's *t* test. *p < 0.05; **p < 0.01; ***p < 0.001. Vinculin was included as a loading control.

The up‐regulatory mechanism of FTO in EBVaGC remains unclear, and we presumed that EBV controlled upstream transcription factors of the FTO promoter through its antigens, thereby increasing FTO translation and protein expression. RNA‐seq was performed on 73 cases of EBVaGC samples from the SYSUCC cohort, and a hallmark enriched pathway analysis prompted that FTO expression was positively correlated with MYC pathway in EBVaGC tumour tissues (Figure S7A–C). Moreover, a previous study stated that MYC, as a transcription factor, was positively regulated by EBNA1,^48^ and we observed that the expression of MYC was notably enhanced in EBVaGC cells compared with EBVnGC cells (Figure 7A). Therefore, to test our hypothesis, an RT‐qPCR assay was applied to show that MYC expression was positively correlated with EBNA1 and FTO expressions in 62 EBVaGC specimens from SYSUCC (Figure 7B). To further verify whether MYC could transcriptionally enhance FTO expression, we next overexpressed MYC and constructed MYC‐silencing EBVaGC cell lines (Figure S7D,E) and observed that high expression of MYC obviously elevated the expression of FTO, while MYC silencing led to the reduced expression of FTO at the protein and mRNA levels (Figure 7C–F). Then, dual‐luciferase promoter activity analysis illustrated that MYC up‐regulation effectively promoted the transcriptional activity of FTO, and the opposite effect occurred with the depletion of MYC in EBVaGC cells (Figure 7G,H). The Jaspar (https://jaspar.genereg.net/) database predicted that MYC was directly bound to the −1364 to −1354 regions of the FTO promoter, which was subsequently verified using a ChIP assay (Figure 7I,J). Additionally, the MYC‐binding sequence on the FTO promoter was deleted, as shown in Figure 7I, and we found that MYC overexpression only up‐regulated the transcriptional activity of the wild‐type FTO promoter but had no significant effect on the mutant FTO promoter (Figure 7K,L). Collectively, the results indicated that MYC increased the transcription and expression of FTO mainly through specifically binding the FTO promoter in EBVaGC cells.

### 3.8 The FTO/FOS m6A regulatory axis also exists in EBVnGC cells

**FIGURE 8:**
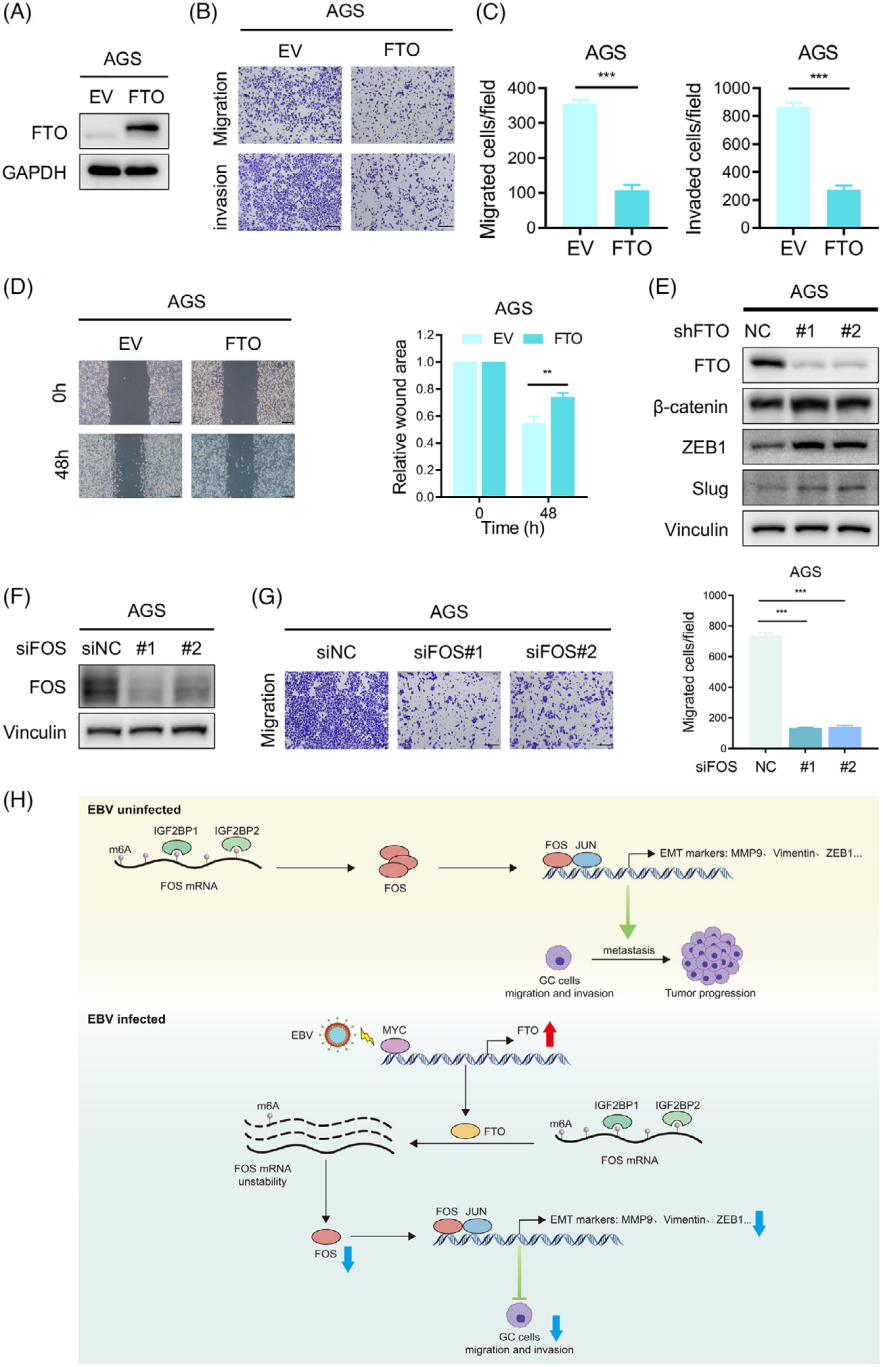
The FTO/FOS m6A regulatory axis also exists in EBVnGC cells. (A) Western blotting assay of FTO protein levels in FTO‐overexpressing (FTO) and empty vector (EV) AGS cells. (B and C) Representative images (B) and quantification (C) of migrated and invaded AGS cells with FTO and EV. Scale bar: 200 µm. (D) Wound healing assays of AGS cells overexpressing FTO and control cells are shown and quantitatively analysed. Scale bar: 200 µm. (E) The protein levels of EMT markers (β‐Catenin, ZEB1, Slug) in AGS cells with FTO silencing were detected by western blotting. (F) Western blotting of FOS expression in FOS knockdown with si#1 and si#2 versus control AGS cells. (G) Images (left) and quantification (right) of the transwell migration assay of AGS cells after FOS deficiency. Scale bar: 200 µm. (H) Proposed working model of the underlying mechanism in this work. The data in (C, D and G) are presented as the means ± SDs. *p*‐Values were determined by Student's *t* test. *p < 0.05; **p < 0.01; ***p < 0.001. GAPDH (A) and Vinculin (E and F) were included as loading controls.

Several studies have revealed that EBVnGC has significantly stronger metastatic potential than EBVaGC.^12^, ^13^, ^49^ As mentioned above, FTO obviously suppressed EBVaGC metastasis in vitro and in vivo, but it was low expressed in EBVnGC cells. Dramatically, in EBVnGC cells, we found that the ectopic expression of FTO restrained cell migration, invasion and the wound healing rate (Figure 8A–D), whereas the protein levels of EMT marker genes were further increased after FTO depletion (Figure 8E). Furthermore, FOS knockdown was expected to successfully inhibit the migratory ability of EBVnGC cells (Figure 8F,G). Altogether, these data showed that there existed the FTO/FOS m6A regulatory axis in EBVnGC, which might provide us with potential therapeutic and prognostic targets for GC.

## 4 DISCUSSION

To date, studies have revealed a variety of eukaryotic mRNA modifications, including adenosine methylation to m6A, N1‐methyladenosine (m1A), and N6,2′‐ o‐dimethyladenosine (m6Am), as well as cytosine methylation to form 5‐methylcytosine and its oxidation product 5‐hydroxymethylcytosine (hm5c).^50^, ^51^ Among those modifications, m6A modification, as the most abundant internal mRNA modification, is a dynamic and reversible process jointly mediated by m6A writers, erasers and readers. In addition, m6A plays a crucial role in affecting RNA metabolism, including splicing, export, translation and decay.^18^, ^52^, ^53^ Accumulating evidence suggests that abnormal m6A modification occurs in multiple types of tumours and can affect tumour progression via modulating cancer‐related biological functions, which has become an emerging research frontier in tumour biology.^19^, ^54^ In this study, we firstly explored the m6A level changes in EBVaGC and EBVnGC cells, and MeRIP‐seq results indicated that the hypomethylated m6A peaks were significantly increased in GC cells infected with EBV. Moreover, an m6A dot blot assay was performed to observe that the m6A modification of EBVaGC cells was obviously reduced compared with that of EBVnGC cells, and further experiments verified that high expression of the m6A demethylase FTO in EBVaGC cells leads to abnormal m6A modification. These findings revealed that increasing FTO expression in EBVaGC cells induced the overall down‐regulation of m6A modification.

FTO, acting as the first identified m6A demethylase, has been demonstrated to be frequently dysregulated and participated in the progression of many tumour types which plays a dual role. Previous studies have reported that FTO plays an oncogenic role in melanoma by reducing RNA degradation of critical melanoma‐promoting genes, including PD‐1 and CXCR4, via an m6A‐YTHDF2‐dependent mechanism^55^ and facilitates bladder cancer tumorigenesis and tumour cell viability through the FTO/MALAT/miR‐384/MAL2 signalling pathway.^56^ In contrast, FTO functions as a tumour suppressor in CRC, which depresses MTA1 expression and reduces its mRNA stability in an m6A‐IGF2BP2‐dependent manner.^57^ Additionally, FTO was reported to inhibit ovarian cancer stem cell self‐renewal and restrain tumour progression in thyroid cancer.^58^, ^59^ These results indicated that the role FTO exerts in different tumours mainly relying on the effort of downstream target genes and their m6A binding proteins. Our study uncovered a significant suppressive role for FTO in EBVaGC metastasis and invasiveness through a series of in vitro and in vivo experiments and showed that FTO expression was associated with better prognosis in GC patients, suggesting that FTO might be a biomarker for predicting the metastasis and outcome of GC patients. Meanwhile, the body weight of all B‐NDG mice with FTO treatment in this work suggested that there was less toxicity profile of the FTO response in animals (Figure S7F–I), which showed a prospective therapy using FTO as a target. In addition, the FTO mutant plasmid had no significance on EBVaGC cell migration ability, which reflected that FTO inhibited EBVaGC cell metastasis depending on its m6A demethylation activity.

The FOS gene family includes the FOS, FOSB, FOSL1 and FOSL2 genes, and these members encode leucine zipper proteins that can dimerise with JUN family proteins (c‐JUN, JUNB, JUND), leading to the formation of the AP‐1 transcription factor complex, which is involved in cell proliferation, differentiation, migration and invasion.^60^, ^61^ For instance, c‐FOS in the inflammatory microenvironment activated by GDF15 promotes CRC invasion and metastasis through modulating EMT genes.^40^ Moreover, one study stated that c‐FOS regulated by the transcription factor c‐Myb increased the EMT molecular phenotype in CRC cells, thus accelerating the malignant progression of CRC.^62^ These data revealed that FOS participated in the regulation of tumour metastasis as an AP‐1 transcription factor subunit. Here, we identified FOS as the key downstream target gene of FTO by performing MeRIP‐seq, qPCR and western blotting assays. In phenotypic experiments, FOS overexpression facilitated EBVaGC cell migration, and FTO suppressed EBVaGC cell metastasis by depressing FOS expression. Furthermore, FTO inhibition and mutant plasmids were used to demonstrate the modulation of FOS expression by FTO in an m6A‐dependent manner, where m6A modification enhanced FOS transcript stability. Our current findings indicated that FTO highly expressed in EBVaGC cells eliminated m6A modification of FOS mRNA through its demethylase activity and naturally induced FOS mRNA decay and inhibited its expression, thereby suppressing EBVaGC cell migration and invasion.

The m6A readers are required to mediate the fate of methylated mRNA. Our data verified that IGF2BP1/2 directly bound to specific m6A modification regions in FOS mRNA. Previous reports have shown that IGF2BPs serve as posttranscriptional fine‐tuners for target mRNA and precisely bind thousands of mRNA transcripts via recognizing the conserved GG (m6A) C sequence. IGF2BPs are involved in regulating RNA processing and metabolism, such as RNA stability, translation and localization. Functionally, contrary to the promoting role of mRNA degradation by YTHDF2, IGF2BPs fortify the stability and storage of m6A‐modified target transcripts in an m6A‐dependent mechanism.^45^, ^46^, ^63^, ^64^, ^65^ Here, we detected that silencing IGF2BP1/2 depressed FOS expression and shortened its mRNA lifespan, meanwhile, IGF2BP1/2 knockdown restrained EBVaGC cell migration in the absence of FTO. These results revealed an underlying FTO‐IGF2BP1/2 m6A regulatory mechanism of FOS expression in EBVaGC and partially accounted for the promoting effect of IGF2BP1/2 on EBVaGC cell metastasis, providing a promising therapeutic strategy for targeting FOS and dampening metastasis in GC.

In summary, our work investigated the tumour suppressive role of FTO and its downstream regulatory mechanism based on aberrant m6A modification in EBVaGC and uncovered that the FTO‐FOS‐IGF2BP1/2 signalling pathway delayed malignant progression in an m6A‐dependent manner. In addition, we found that MYC induced FTO expression in the case of EBV infection, which accounted for the lower metastasis rate in EBVaGC. Furthermore, it is possible to extend the FTO‐FOS‐IGF2BP1/2 axis as biomarkers to all patients with GC, regardless of their EBV infection status, and detecting the expression of this biomarker panel could predict the prognosis of GC patients. However, there are some limitations in our study. The regulatory mode of FTO‐FOS‐IGF2BP1/2‐m6A is one of the mechanisms that explains the declining metastasis in EBVaGC, and other potential downstream target genes of FTO need to be further verified. In general, the FTO‐FOS‐IGF2BP1/2 pathway indicated a promising therapeutic strategy for GC patients, which we will focus on exploring in the future (Figure 8H).

## AUTHOR CONTRIBUTIONS

*Conceptualization*: Zhao‐lei Zeng, Zhi‐Xiang Zuo and Yun‐Yun Xu. *Methodology*: Yun‐Yun Xu, Ting Li, Ao Shen, Xiao‐Qiong Bao, Jin‐Fei Lin, Li‐Zhen Guo, Dan‐Yun Ruan and Qi‐Hua Zhang. *Bioinformatics analysis*: Xiao‐Qiong Bao, Ao Shen and Qi Meng. *Investigation*: Yun‐Yun Xu, Ting Li, Jin‐Fei Lin, Li‐Zhen Guo, Dan‐Yun Ruan and Zhang QH. *Writing—original draft*: Yun‐Yun Xu. *Writing—review and editing*: Zhao‐lei Zeng, Zhi‐Xiang Zuo, Yun‐Yun Xu, Ting Li, Ao Shen and Xiao‐Qiong Bao. *Funding acquisition*: Zhao‐lei Zeng, Zhi‐Xiang Zuo and Dan‐Yun Ruan. *Supervision*: Zhao‐lei Zeng and Zhi‐Xiang Zuo. All authors read and approved the final manuscript.

## CONFLICT OF INTEREST STATEMENT

All the authors declared that they have no competing interests.

## FUNDING INFORMATION

National Natural Science Foundation of China, Grant Numbers: 82072612 and 82203481; CAMS Innovation Fund for Medical Sciences (CIFMS), Grant Number: 2019‐I2M‐5‐036; Guangdong Basic and Applied Basic Research Foundation, Grant Number: 2021B1515020108; Program for Guangdong Introducing Innovative and Entrepreneurial Teams, Grant Number: 2017ZT07S096

## ETHICS STATEMENT

All experiments were approved by the Institutional Research Ethics Committee of Sun Yat‐sen University Cancer Center.

## Supporting information

Supporting Information

## Data Availability

All data supporting the findings of this work are included in this article and the supplementary materials files. The MeRIP‐seq data (GSA‐Human: HRA004157, https://ngdc.cncb.ac.cn/gsa‐human/s/7DAT97Fv) and RNA‐seq data (GSA‐Human: HRA005280, https://ngdc.cncb.ac.cn/gsa‐human/s/R1mJeTDt) have been deposited in the Genome Sequence Archive in National Genomics Data Center, China National Center for Bioinformation/Beijing Institute of Genomics, Chinese Academy of Sciences that are publicly accessible at https://ngdc.cncb.ac.cn/gsa‐human.

## References

[1] Sung H , Ferlay J , Siegel RL , et al. Global cancer statistics 2020: GLOBOCAN estimates of incidence and mortality worldwide for 36 cancers in 185 countries. CA Cancer J Clin. 2021;71(3):209‐249. 33538338 10.3322/caac.21660

[2] Cancer Genome Atlas Research Network . Comprehensive molecular characterization of gastric adenocarcinoma. Nature. 2014;513(7517):202‐209. 25079317 10.1038/nature13480 PMC4170219

[3] Rocken C . Molecular classification of gastric cancer. Expert Rev Mol Diagn. 2017;17(3):293‐301. 28118758 10.1080/14737159.2017.1286985

[4] Chia NY , Tan P . Molecular classification of gastric cancer. Ann Oncol. 2016;27(5):763‐769. 26861606 10.1093/annonc/mdw040

[5] Murata T . Lifecycles of EBV and cancer. Uirusu. 2014;64(1):95‐104. 25765985 10.2222/jsv.64.95

[6] Ressing ME , van Gent M , Gram AM , et al. Immune evasion by Epstein‐Barr virus. Curr Top Microbiol Immunol. 2015;391:355‐381. 26428381 10.1007/978-3-319-22834-1_12

[7] Kerr JR . Epstein‐Barr virus (EBV) reactivation and therapeutic inhibitors. J Clin Pathol. 2019;72(10):651‐658. 31315893 10.1136/jclinpath-2019-205822

[8] Kanda T . EBV‐encoded latent genes. Adv Exp Med Biol. 2018;1045:377‐394. 29896676 10.1007/978-981-10-7230-7_17

[9] Shinozaki‐Ushiku A , Kunita A , Fukayama M . Update on Epstein‐Barr virus and gastric cancer (review). Int J Oncol. 2015;46(4):1421‐1434. 25633561 10.3892/ijo.2015.2856

[10] Lee JH , Kim SH , Han SH , et al. Clinicopathological and molecular characteristics of Epstein‐Barr virus‐associated gastric carcinoma: a meta‐analysis. J Gastroenterol Hepatol. 2009;24(3):354‐365. 19335785 10.1111/j.1440-1746.2009.05775.x

[11] Morales‐Sanchez A , Fuentes‐Panana EM . Epstein‐Barr virus‐associated gastric cancer and potential mechanisms of oncogenesis. Curr Cancer Drug Targets. 2017;17(6):534‐554. 27677953 10.2174/1568009616666160926124923

[12] Yang J , Liu Z , Zeng B , Hu G , Gan R . Epstein‐Barr virus‐associated gastric cancer: a distinct subtype. Cancer Lett. 2020;495:191‐199. 32979463 10.1016/j.canlet.2020.09.019

[13] Nishikawa J , Iizasa H , Yoshiyama H , et al. Clinical importance of Epstein(‐)Barr virus‐associated gastric cancer. Cancers (Basel). 2018;10(6):167. 29843478 10.3390/cancers10060167 PMC6024931

[14] Qiu MZ , He CY , Lu SX , et al. Prospective observation: clinical utility of plasma Epstein‐Barr virus DNA load in EBV‐associated gastric carcinoma patients. Int J Cancer. 2020;146(1):272‐280. 31162842 10.1002/ijc.32490

[15] Xu Y , Shen A , Zeng Z . A potential EBV‐related classifier is associated with the efficacy of immunotherapy in gastric cancer. Transl Cancer Res. 2022;11(7):2084‐2096. 35966300 10.21037/tcr-22-461 PMC9372194

[16] Chen JN , He D , Tang F , Shao CK . Epstein‐Barr virus‐associated gastric carcinoma: a newly defined entity. J Clin Gastroenterol. 2012;46(4):262‐271. 22392024 10.1097/MCG.0b013e318249c4b8

[17] Sun T , Wu R , Ming L . The role of m6A RNA methylation in cancer. Biomed Pharmacother. 2019;112:108613. 30784918 10.1016/j.biopha.2019.108613

[18] He L , Li H , Wu A , et al. Functions of N6‐methyladenosine and its role in cancer. Mol Cancer. 2019;18(1):176. 31801551 10.1186/s12943-019-1109-9 PMC6892141

[19] Ma S , Chen C , Ji X , et al. The interplay between m6A RNA methylation and noncoding RNA in cancer. J Hematol Oncol. 2019;12(1):121. 31757221 10.1186/s13045-019-0805-7 PMC6874823

[20] Huang H , Weng H , Chen J . m(6)A modification in coding and non‐coding RNAs: roles and therapeutic implications in cancer. Cancer Cell. 2020;37(3):270‐288. 32183948 10.1016/j.ccell.2020.02.004 PMC7141420

[21] Lan Q , Liu PY , Haase J , et al. The critical role of RNA m(6)A methylation in cancer. Cancer Res. 2019;79(7):1285‐1292. 30894375 10.1158/0008-5472.CAN-18-2965

[22] An Y , Duan H . The role of m6A RNA methylation in cancer metabolism. Mol Cancer. 2022;21(1):14. 35022030 10.1186/s12943-022-01500-4 PMC8753874

[23] Chen XY , Zhang J , Zhu JS . The role of m(6)A RNA methylation in human cancer. Mol Cancer. 2019;18(1):103. 31142332 10.1186/s12943-019-1033-z PMC6540575

[24] Li T , Hu PS , Zuo Z , et al. METTL3 facilitates tumor progression via an m(6)A‐IGF2BP2‐dependent mechanism in colorectal carcinoma. Mol Cancer. 2019;18(1):112. 31230592 10.1186/s12943-019-1038-7 PMC6589893

[25] Ma JZ , Yang F , Zhou CC , et al. METTL14 suppresses the metastatic potential of hepatocellular carcinoma by modulating N(6) ‐methyladenosine‐dependent primary MicroRNA processing. Hepatology. 2017;65(2):529‐543. 27774652 10.1002/hep.28885

[26] Deng X , Su R , Weng H , et al. RNA N(6)‐methyladenosine modification in cancers: current status and perspectives. Cell Res. 2018;28(5):507‐517. 29686311 10.1038/s41422-018-0034-6 PMC5951805

[27] Jia G , Fu Y , Zhao X , et al. N6‐methyladenosine in nuclear RNA is a major substrate of the obesity‐associated FTO. Nat Chem Biol. 2011;7(12):885‐887. 22002720 10.1038/nchembio.687 PMC3218240

[28] Cui YH , Yang S , Wei J , et al. Autophagy of the m(6)A mRNA demethylase FTO is impaired by low‐level arsenic exposure to promote tumorigenesis. Nat Commun. 2021;12(1):2183. 33846348 10.1038/s41467-021-22469-6 PMC8041927

[29] Zou D , Dong L , Li C , et al. The m(6)A eraser FTO facilitates proliferation and migration of human cervical cancer cells. Cancer Cell Int. 2019;19:321. 31827395 10.1186/s12935-019-1045-1 PMC6888952

[30] Karin M , Liu Z , Zandi E . AP‐1 function and regulation. Curr Opin Cell Biol. 1997;9(2):240‐246. 9069263 10.1016/s0955-0674(97)80068-3

[31] Shaulian E , Karin M . AP‐1 as a regulator of cell life and death. Nat Cell Biol. 2002;4(5):E131‐E136. 11988758 10.1038/ncb0502-e131

[32] Sundqvist A , Vasilaki E , Voytyuk O , et al. TGFbeta and EGF signaling orchestrates the AP‐1‐ and p63 transcriptional regulation of breast cancer invasiveness. Oncogene. 2020;39(22):4436‐4449. 32350443 10.1038/s41388-020-1299-z PMC7253358

[33] Zhu G , Cheng Z , Huang Y , et al. MyD88 mediates colorectal cancer cell proliferation, migration and invasion via NFkappaB/AP1 signaling pathway. Int J Mol Med. 2020;45(1):131‐140. 31746347 10.3892/ijmm.2019.4390 PMC6889924

[34] Huang Q , Lan F , Wang X , et al. IL‐1beta‐induced activation of p38 promotes metastasis in gastric adenocarcinoma via upregulation of AP‐1/c‐fos, MMP2 and MMP9. Mol Cancer. 2014;13:18. 24479681 10.1186/1476-4598-13-18 PMC3937117

[35] Ma F , Wang H , Liu K , Wang Z , Chen S . CSN6 inhibition suppresses pancreatic adenocarcinoma metastasis via destabilizing the c‐Fos protein. Exp Cell Res. 2020;391(1):112004. 32289284 10.1016/j.yexcr.2020.112004

[36] Li P , Lin Z , Liu Q , et al. Enhancer RNA SLIT2 inhibits bone metastasis of breast cancer through regulating P38 MAPK/c‐Fos signaling pathway. Front Oncol. 2021;11:743840. 34722297 10.3389/fonc.2021.743840 PMC8554345

[37] Liu ZG , Jiang G , Tang J , et al. c‐Fos over‐expression promotes radioresistance and predicts poor prognosis in malignant glioma. Oncotarget. 2016;7(40):65946‐65956. 27602752 10.18632/oncotarget.11779 PMC5323205

[38] Jeong YJ , Cho HJ , Chung FL , et al. Isothiocyanates suppress the invasion and metastasis of tumors by targeting FAK/MMP‐9 activity. Oncotarget. 2017;8(38):63949‐63962. 28969043 10.18632/oncotarget.19213 PMC5609975

[39] Yang B , Zhang D , Qian J , Cheng Y . Chelerythrine suppresses proliferation and metastasis of human prostate cancer cells via modulating MMP/TIMP/NF‐kappaB system. Mol Cell Biochem. 2020;474(1‐2):199‐208. 32737771 10.1007/s11010-020-03845-0

[40] Ding Y , Hao K , Li Z , et al. c‐Fos separation from Lamin A/C by GDF15 promotes colon cancer invasion and metastasis in inflammatory microenvironment. J Cell Physiol. 2020;235(5):4407‐4421. 31613004 10.1002/jcp.29317

[41] Huang Y , Su R , Sheng Y , et al. Small‐molecule targeting of oncogenic FTO demethylase in acute myeloid leukemia. Cancer Cell. 2019;35(4):677‐691.e10. 30991027 10.1016/j.ccell.2019.03.006 PMC6812656

[42] Van Der Werf I , Jamieson C . The yin and yang of RNA methylation: an imbalance of erasers enhances sensitivity to FTO demethylase small‐molecule targeting in leukemia stem cells. Cancer Cell. 2019;35(4):540‐541. 30991023 10.1016/j.ccell.2019.03.011

[43] Zhao Z , Zeng J , Guo Q , et al. Berberine suppresses stemness and tumorigenicity of colorectal cancer stem‐like cells by inhibiting m(6)A methylation. Front Oncol. 2021;11:775418. 34869024 10.3389/fonc.2021.775418 PMC8634032

[44] Yang Y , Hsu PJ , Chen YS , Yang YG . Dynamic transcriptomic m(6)A decoration: writers, erasers, readers and functions in RNA metabolism. Cell Res. 2018;28(6):616‐624. 29789545 10.1038/s41422-018-0040-8 PMC5993786

[45] Zhao Y , Shi Y , Shen H , Xie W . m(6)A‐binding proteins: the emerging crucial performers in epigenetics. J Hematol Oncol. 2020;13(1):35. 32276589 10.1186/s13045-020-00872-8 PMC7146974

[46] Huang H , Weng H , Sun W , et al. Recognition of RNA N(6)‐methyladenosine by IGF2BP proteins enhances mRNA stability and translation. Nat Cell Biol. 2018;20(3):285‐295. 29476152 10.1038/s41556-018-0045-z PMC5826585

[47] Tong J , Flavell RA , Li HB . RNA m(6)A modification and its function in diseases. Front Med. 2018;12(4):481‐489. 30097961 10.1007/s11684-018-0654-8

[48] Owen TJ , O'Neil JD , Dawson CW , et al. Epstein‐Barr virus‐encoded EBNA1 enhances RNA polymerase III‐dependent EBER expression through induction of EBER‐associated cellular transcription factors. Mol Cancer. 2010;9:241. 20843307 10.1186/1476-4598-9-241 PMC2945964

[49] Yau TO , Tang CM , Yu J . Epigenetic dysregulation in Epstein‐Barr virus‐associated gastric carcinoma: disease and treatments. World J Gastroenterol. 2014;20(21):6448‐6456. 24914366 10.3748/wjg.v20.i21.6448 PMC4047330

[50] Roundtree IA , Evans ME , Pan T , He C . Dynamic RNA modifications in gene expression regulation. Cell. 2017;169(7):1187‐1200. 28622506 10.1016/j.cell.2017.05.045 PMC5657247

[51] Jia G , Fu Y , He C . Reversible RNA adenosine methylation in biological regulation. Trends Genet. 2013;29(2):108‐115. 23218460 10.1016/j.tig.2012.11.003 PMC3558665

[52] Liu J , Harada BT , He C . Regulation of gene expression by N(6)‐methyladenosine in cancer. Trends Cell Biol. 2019;29(6):487‐499. 30940398 10.1016/j.tcb.2019.02.008 PMC6527461

[53] Zhao BS , Roundtree IA , He C . Post‐transcriptional gene regulation by mRNA modifications. Nat Rev Mol Cell Biol. 2017;18(1):31‐42. 27808276 10.1038/nrm.2016.132 PMC5167638

[54] Zhang N , Zuo Y , Peng Y , Zuo L . Function of N6‐methyladenosine modification in tumors. J Oncol. 2021;2021:6461552. 34858499 10.1155/2021/6461552 PMC8632389

[55] Yang S , Wei J , Cui YH , et al. m(6)A mRNA demethylase FTO regulates melanoma tumorigenicity and response to anti‐PD‐1 blockade. Nat Commun. 2019;10(1):2782. 31239444 10.1038/s41467-019-10669-0 PMC6592937

[56] Tao L , Mu X , Chen H , et al. FTO modifies the m6A level of MALAT and promotes bladder cancer progression. Clin Transl Med. 2021;11(2):e310. 33634966 10.1002/ctm2.310 PMC7851431

[57] Ruan DY , Li T , Wang YN , et al. FTO downregulation mediated by hypoxia facilitates colorectal cancer metastasis. Oncogene. 2021;40(33):5168‐5181. 34218271 10.1038/s41388-021-01916-0 PMC8376648

[58] Huang H , Wang Y , Kandpal M , et al. FTO‐dependent N (6)‐methyladenosine modifications inhibit ovarian cancer stem cell self‐renewal by blocking cAMP signaling. Cancer Res. 2020;80(16):3200‐3214. 32606006 10.1158/0008-5472.CAN-19-4044 PMC7442742

[59] Tian R , Zhang S , Sun D , et al. M6A demethylase FTO plays a tumor suppressor role in thyroid cancer. DNA Cell Biol. 2020;39(12):2184‐2193. 10.1089/dna.2020.5956 33054406

[60] Tsiambas E , Mastronikolis N , Fotiades PP , et al. c‐Jun/c‐Fos complex in laryngeal squamous cell carcinoma. J BUON. 2020;25(2):618‐620. 32521843

[61] Milde‐Langosch K . The Fos family of transcription factors and their role in tumourigenesis. Eur J Cancer. 2005;41(16):2449‐2461. 16199154 10.1016/j.ejca.2005.08.008

[62] Qu X , Yan X , Kong C , et al. c‐Myb promotes growth and metastasis of colorectal cancer through c‐fos‐induced epithelial‐mesenchymal transition. Cancer Sci. 2019;110(10):3183‐3196. 31338937 10.1111/cas.14141 PMC6778643

[63] Bell JL , Wachter K , Muhleck B , et al. Insulin‐like growth factor 2 mRNA‐binding proteins (IGF2BPs): post‐transcriptional drivers of cancer progression? Cell Mol Life Sci. 2013;70(15):2657‐2675. 23069990 10.1007/s00018-012-1186-z PMC3708292

[64] Huang X , Zhang H , Guo X , et al. Insulin‐like growth factor 2 mRNA‐binding protein 1 (IGF2BP1) in cancer. J Hematol Oncol. 2018;11(1):88. 29954406 10.1186/s13045-018-0628-y PMC6025799

[65] Du QY , Zhu ZM , Pei DS . The biological function of IGF2BPs and their role in tumorigenesis. Invest New Drugs. 2021;39(6):1682‐1693. 34251559 10.1007/s10637-021-01148-9

