# Supplementary material for: FTO up‐regulation induced by MYC suppresses tumour progression in Epstein‒Barr virus‐associated gastric cancer
Source: Clin Transl Med. 2023 Dec 11;13(12):e1505. doi: 10.1002/ctm2.1505 (PMC10713874; doi:10.1002/ctm2.1505)
Supplement: Supplementary file 1 — Supporting Information [file CTM2-13-e1505-s001.docx]

**SUPPLEMENTARY MATERIAL**

**RNA isolation and real-time quantitative polymerase chain reaction (RT‒qPCR)**

RNA isolation procedures and RT‒qPCR assays were performed as previously reported^1^. The relative mRNA expression was calculated using the 2-ΔCt or 2-ΔΔCt method, and β-Actin was used as an endogenous control. The primers applied in this work were synthesized by TSINGKE Biological Technology (Guangzhou, China) and are listed in Supplementary Table S3.

**Western blotting**

Protein was obtained using RIPA buffer (P0013, Beyotime) containing protease and phosphatase inhibitors (78442, Thermo Fisher Scientific), and protein concentrations were then measured using a BCA protein assay kit (23227, Invitrogen). All samples were diluted to equal protein concentrations and subsequently separated by SDS‒PAGE and transferred onto a PVDF membrane (IPVH00010, Merck Millipore). The membrane was blocked with 5% nonfat milk and incubated with the respective primary antibodies at 4 °C overnight. After incubation with horseradish peroxidase-coupled secondary antibodies (ZB-2301, ZSGB-BIO) at room temperature for 1 hour, the bands were visualized using chemiluminescence assays (34096, Invitrogen). Detailed information on the antibodies used is listed in Supplementary Table S5.

**Wound healing assay**

Cells were seeded in 6-well plates (TCP011006, Jet), which were labelled with horizontal lines on the back using a marker pen. When the cells reached the desired confluence, a pipette tip was conducted to scratch a wound perpendicular to the marked line across the center of the well. The cells were then gently washed with PBS, and serum-free medium was added. Wound healing was recorded at 0, 24, and 48 hours using a microscope. ImageJ software was utilized to calculate the healing area of cell scratches.

**Cell migration and invasion assays**

The cell migration assay was determined using a 24-well Transwell chamber system (353097, Corning), and cell invasion was performed using a BioCoat Matrigel Invasion Chamber (354480, Corning). In brief, cells (0.5-1×10^5^) in 200 μL of serum-free medium were seeded into the upper chamber, and the lower chamber was filled with 600 μL of 20% FBS medium as a chemoattractant. After 16-18 hours, migrated or invaded cells were fixed with methanol, stained with 0.1% crystal violet, and counted by a microscope.

**Cell proliferation assay**

Cell growth was determined by performing the MTS assay. GC cells (1×10^3^ cells/well) of different groups were seeded in 96-well plates (TCP011096, Jet), and at different time points, the spectrophotometric absorbance of 96-well plates was measured at 490 nm using a Synergy™ Multi-Mode Microplate Reader (Biotek).

**Immunohistochemistry (IHC)**

Paraffin-embedded sections were deparaffinized and rehydrated, and then, the endogenous peroxidase activity of sections was blocked with 3% H_2_O_2_ for 10 min. The samples were heated at a subboiling temperature in sodium citrate buffer or EDTA for 10 min using a pressure cooker for antigen retrieval, and they were blocked with 10% FBS at room temperature for 30 min. The sections were then incubated with primary antibody at 37 °C for 1 hour and treated with secondary antibody for 30 min at room temperature. A Dako REAL™ EnVision™ Detection System (K5007, Copenhagen, Denmark) was used to detect target protein expression, and counterstaining was conducted using haematoxylin. The staining intensities of the sections were assessed on a scale of 0 to 3 (0, negative staining; 1, weak staining; 2, moderate staining; 3, strong staining), and the final IHC score was calculated by multiplying the staining intensity scores by the percentage of positively stained cells (0 to 100%). The antibodies used in IHC are listed in Supplementary Table S5.

**Methylated RNA immunoprecipitation sequencing (MeRIP-seq)**

MeRIP-seq was mainly supported by LC-Bio Technology (Hangzhou, China). Total RNA was extracted and purified using TRIzol reagent (15596018, Invitrogen) as described above and then quantified using a NanoDrop ND-1000 (Wilmington, DE, USA). The RNA integrity was evaluated by a Bioanalyzer 2100 (Agilent, CA, USA) with RIN number >7.0 and OD260/280>1.8 and also verified using agarose gel electrophoresis assays. Poly(A) RNA was purified from 50 μg of total RNA using Dynabeads Oligo (dT) (25-61005, Thermo Fisher) and sheared to approximately 100 nt in length by a Magnesium RNA Fragmentation Module (E6150S, NEB), and 1/10 of the fragmented pool was saved as an input control for further sequencing. The remaining RNA was subsequently incubated with anti-m6A antibody (202003, Synaptic Systems) for 1 hour at 4 °C and mixed with prewashed Pierce™ Protein A/G Magnetic Beads (88803, Thermo Scientific) in immunoprecipitation buffer at 4 °C overnight. MeRIP-seq library preparation and sequencing on an Illumina Novaseq™ 6000 system were performed by LC-Bio Technology (Hangzhou, China).

MeRIP-seq analysis: MeRIPseqPipe^2^ was used to analyze MeRIP-seq data. For detail, FastQC (https://www.bioinformatics.babraham.ac.uk/projects/fastqc/) and fastp^3^ were employed to perform quality control and preprocessing. Then the preprocessed data were aligned to the reference genome (hg38) using STAR^4^. featureCounts^5^ was used to generate gene counts. We used MACS2^6^ (-q 0.01 --keep-dup 5) and MeTPeak^7^ to identify m6A enriched peaks. To ensure a high veracity of peak calling results, RobustRankAggreg (RRA) method^8^ was used to generate consensus peaks from the above two methods. The m6A peak annotation was performed with the human annotation file (GENCODE, version 25) downloaded from the GENCODE database (https://www.gencodegenes.org/) using our custom Perl scripts. In addition, the sequence motifs on m6A peaks were identified by HOMER^9^. For m6A quantification, we calculated the RPKM value for each m6A peak based on reads count calculated using Multicov from BEDTools^10^, and then use the radio of IP RPKM (with adding 1) and INPUT RPKM (with adding 1) for each m6A peak to represent the methylation level of each m6A peak. DESeq2^11^ was used to identify differentially expressed genes. The KEGG pathway analysis of the shared genes were conducted by R package ‘clusterProfiler’^12^. And the R package ‘GSVA’^13^ was used to do single-sample gene set enrichment analysis (ssGSEA).

**RNA sequencing (RNA-seq)**

RNA-seq of EBVaGC patient samples was conducted by HaploX Medical Laboratory Co., Ltd (Jiangxi, China). Total RNA was first extracted from human EBVaGC tumour tissues using TRIzol reagent (15596018, Invitrogen) according to the manufacturer’s protocols, and the quality and purity of RNA were assessed by a NanoDrop™ One/OneC spectrophotometer (Thermo Scientific, MA, USA) and Qubit RNA BR Assay Kit (Q10211, Invitrogen). Agilent 4200 TapeStation system (Agilent, CA, USA) was used to identify RNA integrity. Subsequently, 1 μg of total RNA was prepared for library construction, and RNA libraries were constructed by a VAHTSTM Total RNA-seq (H/M/R) Library Prep Kit for Illumina (NR603, Vazyme). Qubit® 3.0 Fluorometer (Thermo Scientific, MA, USA) was used to measure the concentration of library and Agilent 4200 TapeStation system was performed to check the distribution of segments in library. Library molar concentration was then measured by QuantStudio 5 qPCR systems (Thermo Scientific, MA, USA). Finally, libraries were sequenced using illumina NovaSeq™ 6000 platform (San Diego, CA, USA) and NovaSeq 6000 S4 Reagent Kit V1.5 (20028312, illumina).

RNA-seq analysis: The RNA-seq data analysis was performed as our methodology described previously^1,14^. Briefly, we used FastQC v0.11.9 (https://github.com/s-andrews/FastQC) and MultiQC v1.0.dev0^15^ to estimate the quality of sequencing data, and fastp v0.23.1 software^3^ was applied to trim the adaptor sequences and reads with low quality. Then we used STAR v2.7.9a^4^ to map the reads to the human hg38 reference genome. RSEM v1.3.3^16^ was performed to quantify the expression of genes. For downstream analyses, TPM values was transformed into log2(TPM + 1). We divided the samples into two groups according to the median expression of FTO, then “limma” R package^17^ was used to perform the differential expression gene (DEGs) analysis. For GSEA, we used the HALLMARK pathways in MSigDB^18^ and “clusterProfiler” R package^12,19^ to calculate the normalized enrichment score (NES), with parameters set as: nPermSimple = 100000, eps = 0 in GSEA function.

**RNA immunoprecipitation (RIP) assay**

RIP assays were performed using a Magna RIP RNA-Binding Protein Immunoprecipitation Kit (17–700, Millipore) following the recommended protocols. Concisely, the magnetic beads respectively coated with anti-IGF2BP1 antibody (ab184305, Abcam), anti-IGF2BP2 antibody (ab128175, Abcam) or rabbit IgG were incubated with the collected cell lysates at 4 °C overnight. After 6 washes and digestion with proteinase K buffer, the precipitated RNA was finally extracted using phenol-chloroform-isoamyl alcohol reagent, and the relative interaction between IGF2BP1/2 and FOS RNA was analysed by qPCR and normalized to the input.

**SUPPLEMENTARY REFERENCE**

1. Xu YY, Shen A, Zeng ZL. A potential EBV-related classifier is associated with the efficacy of immunotherapy in gastric cancer. *Transl Cancer Res*. 2022;11(7):2084-2096

2. Bao X, Zhu K, Liu X et al. MeRIPseqPipe: an integrated analysis pipeline for MeRIP-seq data based on Nextflow. *Bioinformatics*. 2022;38(7):2054-2056

3. Chen S, Zhou Y, Chen Y, Gu J. fastp: an ultra-fast all-in-one FASTQ preprocessor. *Bioinformatics*. 2018;34(17):i884-i890

4. Dobin A, Davis CA, Schlesinger F et al. STAR: ultrafast universal RNA-seq aligner. *Bioinformatics*. 2013;29(1):15-21

5. Liao Y, Smyth GK, Shi W. featureCounts: an efficient general purpose program for assigning sequence reads to genomic features. *Bioinformatics*. 2014;30(7):923-30

6. Zhang Y, Liu T, Meyer CA et al. Model-based analysis of ChIP-Seq (MACS). *Genome Biol*. 2008;9(9):R137

7. Cui X, Meng J, Zhang S, Chen Y, Huang Y. A novel algorithm for calling mRNA m6A peaks by modeling biological variances in MeRIP-seq data. *Bioinformatics*. 2016;32(12):i378-i385

8. Kolde R, Laur S, Adler P, Vilo J. Robust rank aggregation for gene list integration and meta-analysis. *Bioinformatics*. 2012;28(4):573-80

9. Heinz S, Benner C, Spann N et al. Simple combinations of lineage-determining transcription factors prime cis-regulatory elements required for macrophage and B cell identities. *Mol Cell*. 2010;38(4):576-89

10. Quinlan AR, Hall IM. BEDTools: a flexible suite of utilities for comparing genomic features. *Bioinformatics*. 2010;26(6):841-2

11. Love MI, Huber W, Anders S. Moderated estimation of fold change and dispersion for RNA-seq data with DESeq2. *Genome Biol*. 2014;15(12):550

12. Yu G, Wang LG, Han Y, He QY. clusterProfiler: an R package for comparing biological themes among gene clusters. *OMICS*. 2012;16(5):284-7

13. Hanzelmann S, Castelo R, Guinney J. GSVA: gene set variation analysis for microarray and RNA-seq data. *BMC Bioinformatics*. 2013;14:7

14. Shen A, Ye Y, Chen F et al. Integrated multi-omics analysis identifies CD73 as a prognostic biomarker and immunotherapy response predictor in head and neck squamous cell carcinoma. *Front Immunol*. 2022;13:969034

15. Ewels P, Magnusson M, Lundin S, Kaller M. MultiQC: summarize analysis results for multiple tools and samples in a single report. *Bioinformatics*. 2016;32(19):3047-8

16. Li B, Dewey CN. RSEM: accurate transcript quantification from RNA-Seq data with or without a reference genome. *BMC Bioinformatics*. 2011;12:323

17. Ritchie ME, Phipson B, Wu D et al. limma powers differential expression analyses for RNA-sequencing and microarray studies. *Nucleic Acids Res*. 2015;43(7):e47

18. Liberzon A, Birger C, Thorvaldsdottir H et al. The Molecular Signatures Database (MSigDB) hallmark gene set collection. *Cell Syst*. 2015;1(6):417-425

19. Wu T, Hu E, Xu S et al. clusterProfiler 4.0: A universal enrichment tool for interpreting omics data. *Innovation (Camb)*. 2021;2(3):100141

**Supplementary Table S1: Correlation analysis for clinicopathologic variables between the EBVaGC and EBVnGC groups among 319 GC patients**

| **Variable** | **EBVaGC** | **EBVnGC** | ***P* value** |
| --- | --- | --- | --- |
| Total | 159 | 160 |  |
| Age, years |  |  | 0.491 |
| ≤60 | 95 | 102 |  |
| > 60 | 64 | 58 |  |
| Gender |  |  | <0.001 |
| Male | 141 | 108 |  |
| Female | 18 | 52 |  |
| T stage |  |  | 0.018 |
| T1 | 20 | 8 |  |
| T2-T4 | 139 | 152 |  |
| N stage |  |  | 0.437 |
| N0 | 42 | 36 |  |
| N1-N3 | 117 | 124 |  |
| M stage |  |  | 0.013 |
| M0 | 139 | 122 |  |
| M1 | 20 | 38 |  |
| TNM stage |  |  | 0.030 |
| I-II | 55 | 75 |  |
| III-IV | 104 | 85 |  |
| Grade |  |  | 0.001 |
| G1-G2 | 69 | 41 |  |
| G3 | 90 | 119 |  |
| Location |  |  | 0.163 |
| Proximal | 74 | 61 |  |
| Distal | 57 | 74 |  |
| Others | 28 | 25 |  |
| Lauren classification |  |  | <0.001 |
| Diffuse | 28 | 27 |  |
| Intestine | 49 | 131 |  |
| Mixed | 82 | 2 |  |
| Vascular invasion |  |  | 0.001 |
| Absent | 76 | 57 |  |
| Present | 76 | 103 |  |
| Unknown | 7 | 0 |  |
| Perineural invasion |  |  | 0.002 |
| Absent | 51 | 34 |  |
| Present | 101 | 126 |  |
| Unknown | 7 | 0 |  |

**Supplementary Table S2: Sequences of siRNAs and shRNAs used in this study**

| **Names** | **Targeted sequences** |
| --- | --- |
| si FTO #1 | GGATGACTCTCATCTCGAA |
| si FTO #2 | GCTGAAATATCCTAAACTA |
| si FOS #1 | GGGATAGCCTCTCTTACTA |
| si FOS #2 | CCTGCAAGATCCCTGATGA |
| si IGF2BP1 #1 | CGAAACACCTGACTCCA |
| si IGF2BP1 #2 | CCTGAAGAAGGTAGAGCAA |
| si IGF2BP2 #1 | CATGCCGCATGATTCTTGA |
| si IGF2BP2 #2 | GAACGAACTGCAGAACTTA |
| si MYC #1 | AGACCUUCAUCAAAAACAU |
| si MYC #2 | GAGCUAAAACGGAGCUUUU |
| sh FTO #1 | GGATGACTCTCATCTCGAA |
| sh FTO #2 | GCTGAAATATCCTAAACTA |

**Supplementary Table S3: Primer sequences used in the PCR analysis**

| **Name** | **Sequence (5’-3’)** |  |
| --- | --- | --- |
| EBNA1-F | GGTCTACTGGCGGTCTATGAT |  |
| EBNA1-R | TTAGGAAGCGTTTCTTGAGCT | |
| LMP2A-F | TTGGGTTCCTAGTGGTTGATAGTC | |
| LMP2A-R | ATGGGGTCCCTAGAAATGGTG | |
| FTO-F | GCTGCTTATTTCGGGACCTG |  |
| FTO-R | AGCCTGGATTACCAATGAG |  |
| ALKBH5-F | CGGCGAAGGCTACACTTACG |  |
| ALKBH5-R | CCACCAGCTTTTGGATCACCA |  |
| METTL3-F | TTGTCTCCAACCTTCCGTAGT |  |
| METTL3-R | CCAGATCAGAGAGGTGGTGTAG |  |
| METTL14-F | AGTGCCGACAGCATTGGTG |  |
| METTL14-R | GGAGCAGAGGTATCATAGGAAGC |  |
| WTAP-F | CTTCCCAAGAAGGTTCGATTGA |  |
| WTAP-R | TCAGACTCTCTTAGGCCAGTTAC |  |
| FOS-F | GGGGCAAGGTGGAACAGTTAT |  |
| FOS-R | CCGCTTGGAGTGTATCAGTCA |  |
| FOS-Region1-F | ATTGCCAACCTGCTGAAGGA |  |
| FOS-Region1-R | TCAGACTCCGGGGTGGCAA |  |
| FOS-Region2-F | CCTTCACCCTGCCTCTCC |  |
| FOS-Region2-R | CAGATAGGTCCATGTCTGGCAC |  |
| FOS-Region3-F | CAAATGCCGCAACCGGAG |  |
| FOS-Region3-R | CCTTCAGCAGGTTGGCAATC |  |
| FOS-Region4-F | CTCTGAGACAGCCCGCTCC |  |
| FOS-Region4-R | GTGGGTGAGCTGAGCGAG |  |
| FOS-Region5-F | AGCTGGTGCATTACAGAGAGGAG |  |
| FOS-Region5-R | ACACTCCATGCGTTTTGCTACAT |  |
| β-Actin-F | TGGATCAGCAAGCAGGAGTA |  |
| β-Actin-R | TCGGCCACATTGTGAACT |  |
| IGF2BP1-F | CGCCACTTATGGACACGCA |  |
| IGF2BP1-R | CCTTAGTGCCCCCTGATGAC |  |
| IGF2BP2-F | TTTACATCGGGAACCTGAGCC |  |
| IGF2BP2-R | GGTAGTCCACGAAGGCGTAG |  |
| CSF1-F | AGACCTCGTGCCAAATTACATT |  |
| CSF1-R | AGGTGTCTCATAGAAAGTTCGGA |  |
| TNFRSF1A-F | TCACCGCTTCAGAAAACCACC |  |
| TNFRSF1A-R | GGTCCACTGTGCAAGAAGAGA |  |
| MAPK3-F | ATGTCATCGGCATCCGAGAC |  |
| MAPK3-R | GGATCTGGTAGAGGAAGTAGCA |  |
| TRADD-F | GCTGTTTGAGTTGCATCCTAGC |  |
| TRADD-R | CCGCACTTCAGATTTCGCA |  |
| MAP2K4-F | TGCAGGGTAAACGCAAAGCA |  |
| MAP2K4-R | CTCCTGTAGGATTGGGATTCAGA |  |
| CREB1-F | ATTCACAGGAGTCAGTGGATAGT |  |
| CREB1-R | CACCGTTACAGTGGTGATGG |  |
| ITCH-F | CCCACGTAGACCAGCATCTG |  |
| ITCH-R | AGAGAGCCTGTACTAGACCCA |  |
| TNFAIP3-F | TTGTCCTCAGTTTCGGGAGAT |  |
| TNFAIP3-R | ACTTCTCGACACCAGTTGAGTT |  |
| MAPK14-F | CCCGAGCGTTACCAGAACC |  |
| MAPK14-R | TCGCATGAATGATGGACTGAAAT |  |
| TRAF3-F | GCGTGTCAAGAGAGCATCGTT |  |
| TRAF3-R | GCAGATGTCCCAGCATTAACT |  |
| FTO-promoter-F | CTGGCCTTGAATTCATGGGTT |  |
| FTO-promoter-R | AGATACAAGCCATGCTTGGTGG |  |
| MYC-F | GGCTCCTGGCAAAAGGTCA |  |
| MYC-R | CTGCGTAGTTGTGCTGATGT |  |

**Supplementary Table S4: The specific sequence of wild-type or m6A motif depletion FOS CDS and 3’-UTR**

| **Vectors** | **Sequence^*^** |
| --- | --- |
| FOS CDS wild-type | ......CTGCTTTGCAGACCGAGATTGCCAACCTGCTGAAGGAGAAGGAAAAACTAGAGTTCATCCTGGCAGCTCACCGACCTGCCTGCAAGATCCCTGATGACCT......CCTTGATCTGACTGGGGGCCTGCCAGAGGTTGCCACCCCGGAGTCTGAGGAGGCCTTCACCCTGCCTCTCCTCAATGACCCTGAGCCCAAGCCCTCAGTGGAACCTGTCAAGAGCATCAGCAGCATGGAGCTGAAGACCGAGCCCTTTGATGACTTCCTGTTCCCAGCATCATCCAGGCCCAGTGGCTCTGAGACAGCCCGCTCCGTGCCAGACATGGACCTATCTGGGTCCTTCTATGCAGCAGACTGGGA......CCCGAGGCTGACTCCTTCCCCAGCTGTGCAGCTGCCCACCGCAAGGGCAGCAGCAGCAATGAGCCTTCCTCTGACTCGCT...... |
| FOS CDS mutation | ......CTGCTTTGC~~AGACC~~GAGATTGCCAACCTGCTGAAGGAGAAGGAA~~AAACT~~AGAGTTCATCCTGGCAGCTCACCGACCTGCCTGCAAGATCCCTGA~~TGACC~~T......CCTTGATC~~TGACT~~GGGGGCCTGCCAGAGGTTGCCACCCCGGAGTCTGAGGAGGCCTTCACCCTGCCTCTCCTCAA~~TGACC~~CTGAGCCCAAGCCCTCAGTG~~GAACC~~TGTCAAGAGCATCAGCAGCATGGAGCTGA~~AGACC~~GAGCCCTTTGA~~TGACT~~TCCTGTTCCCAGCATCATCCAGGCCCAGTGGCTCTG~~AGACA~~GCCCGCTCCGTGCC~~AGACA~~T~~GGACC~~TATCTGGGTCCTTCTATGCAGC~~AGACT~~GGGA......CCCGAGGC~~TGACT~~CCTTCCCCAGCTGTGCAGCTGCCCACCGCAAGGGCAGCAGCAGCAATGAGCCTTCCTC~~TGACT~~CGCT...... |
| FOS 3’-UTR wild-type | ......CCCTAGAGGGTTCCTGTAGACCTAGGGAGGACCTTATCTGTGCGTGAAACACACCAGGCTGTGGGCCTCAAGGACTTGAAAGCATCCATGTGTGGACTCAAGTCCTTACCTCTTCCGGAGATGTAGCAAAACG...... |
| FOS 3'-UTR  mutation | ......CCCTAGAGGGTTCCTGTAGACCTAGGGAGGACCTTATCTGTGCGTGAAACACACCAGGCTGTGGGCCTCAA~~GGACT~~TGAAAGCATCCATGTGT~~GGACT~~CAAGTCCTTACCTCTTCCGGAGATGTAGCAAAACG...... |
|  | *The indicated sequence was cloned to the vectors, and the m6A modification sites are highlighted. |

**Supplementary Table S5: Antibodies for Western blotting,** **IHC, and immunoprecipitation in this study**

| **Antibodies** | **Dilution** | **Company/Catalogue** |
| --- | --- | --- |
| FTO | WB (1:1000), IHC (1:500) | Abcam/ab124892 |
| ALKBH5 | WB (1:1000) | Abcam/ab69325 |
| METTL3 | WB (1:1000) | Abcam/ab195352 |
| FOS | WB (1:1000), IHC (1:4000) | Abcam/ab222699 |
| Vinculin | WB (1:1000) | Proteintech/26520-1-AP |
| ZEB-1 | WB (1:1000) | CST#3396 |
| Slug | WB (1:1000) | CST#9585 |
| β-Catenin | WB (1:1000) | CST#8480 |
| MMP9 | WB (1:1000) | Proteintech/10375-2-AP |
| Vimentin | WB (1:1000) | CST#5741 |
| m6A antibody | WB (1:1000) | Synaptic Systems/202003 |
| β-Actin | WB (1:5000) | Sigma/A5441 |
| GAPDH | WB (1:1000) | CST#2118S |
| Flag | WB (1:1000) | CST#14793S |
| YTHDF1 | WB (1:1000) | Proteintech/17479-1-AP |
| YTHDF2 | WB (1:1000) | Proteintech/24744-1-AP |
| YTHDF3 | WB (1:1000) | Abcam/ab220161 |
| IGF2BP1 | WB (1:1000), IP (1:30) | Abcam/ab184305 |
| IGF2BP2 | WB (1:1000), IP (20 µg/mg) | Abcam/ab128175 |
| IGF2BP3 | WB (1:1000) | Proteintech/14642-1-AP |
| YTHDC1 | WB (1:1000) | Abcam/ab220159 |
| YTHDC2 | WB (1:1000) | Abcam/ab220160 |
| MYC | WB (1:1000), ChIP (1:100) | CST#18583S |

**Supplementary Figure legend**


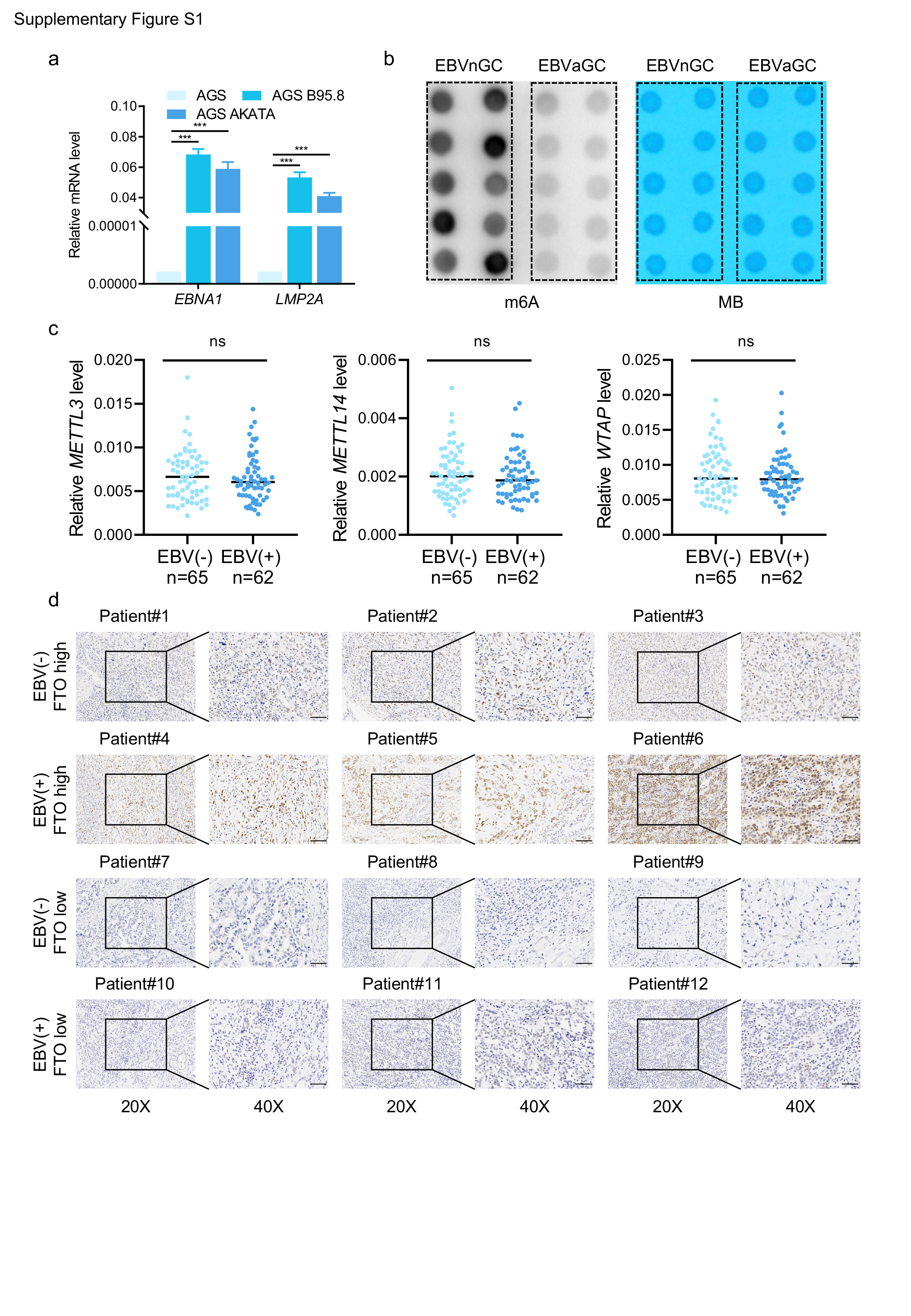


**Supplementary Figure S1.**

**FTO is upregulated in EBVaGC and associated with good prognosis. a** Real-time PCR analysis of EBV latent infection genes in EBV-negative and EBV-positive GC cell lines. **b** RNA m6A dot blot assays using total RNA from 10 cases of EBVnGC and 10 cases of EBVaGC specimens. Methylene blue (MB) staining served as a loading control. **c** RT-qPCR assays of m6A writers (METTL3, METTL14 and WTAP) transcript expression in EBVaGC (n = 62) and EBVnGC tumour tissues (n = 65) from the Sun Yat-sen University Cancer Center (SYSUCC). **d** Representative immunohistochemistry (IHC) images of FTO expression in EBVaGC (n = 159) and EBVnGC tumour samples (n = 160) from SYSUCC. The FTO level was categorized as “high” and “low” based on the median score (median score = 95) in EBVaGC tumour tissues and the median score (median score = 30) in EBVnGC tumour tissues. Scale bar: 50 μm.


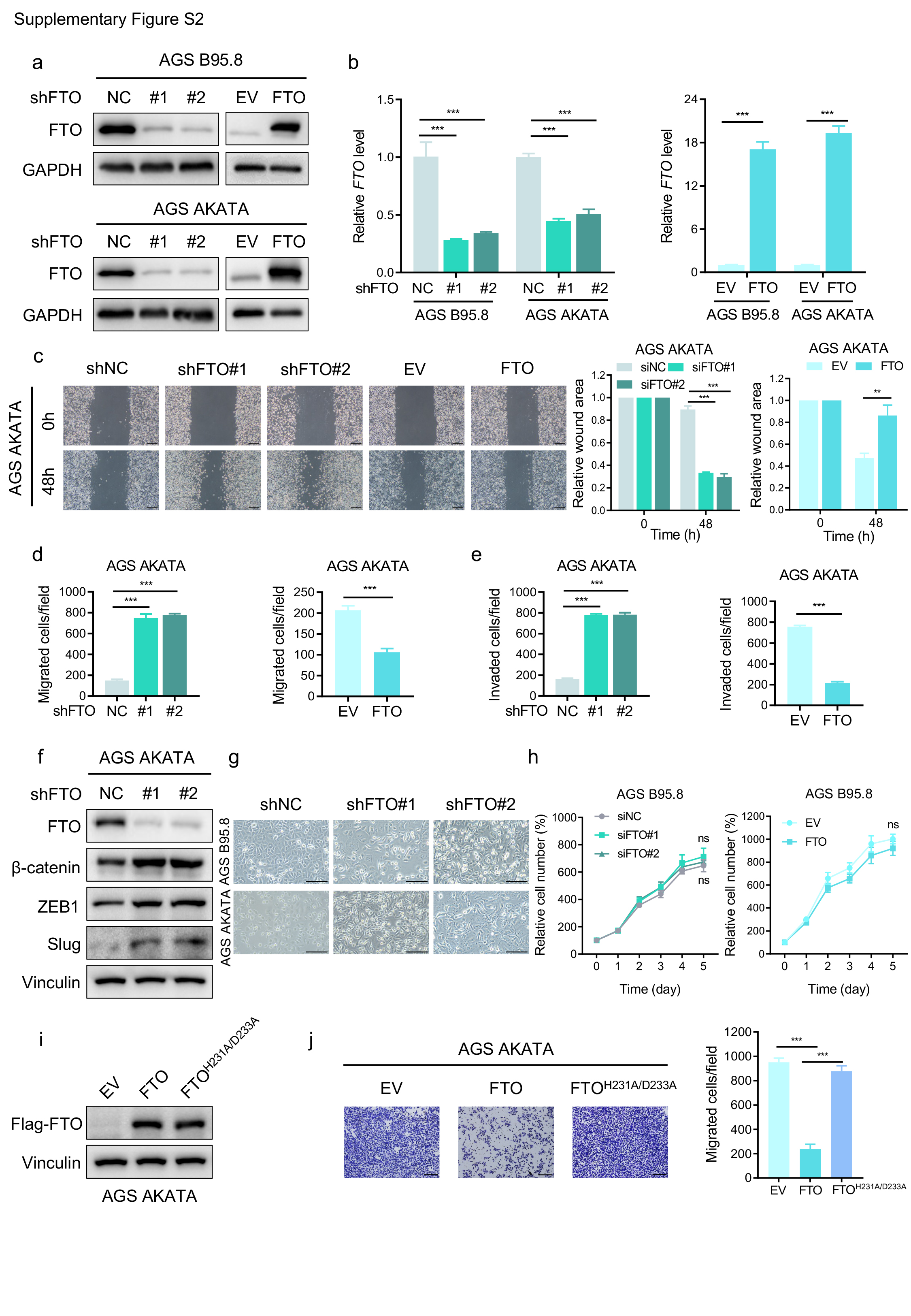


**Supplementary Figure S2.**

**FTO restrains EBVaGC cell migration and invasion *in vitro*. a**, **b** Western blotting (**a**) and RT‒qPCR (**b**) assays of FTO silencing with sh#1 and sh#2 versus normal control (NC) and FTO-overexpressing (FTO) efficiencies versus the empty vector (EV) in EBVaGC cells. **c** Wound healing assays of AGS AKATA cells with FTO knockdown and FTO overexpression were recorded and quantitatively analysed. Scale bar: 200 μm. **d** Quantification of cell migration assays of FTO-knockdown and FTO-overexpressing AGS AKATA cells. **e** Quantification of the cell invasion assay of FTO-knockdown and FTO-overexpressing AGS AKATA cells. **f** The protein levels of EMT markers (β-Catenin, ZEB1, Slug) in AGS AKATA cells after FTO downregulation were measured by immunoblotting. **g** The cell morphology showing that FTO knockdown with sh#1 and sh#2 versus normal control in EBVaGC cells. Scale bar: 200 μm. **h** The MTT assay was performed to determine AGS B95.8 cell growth after FTO silencing (left) and FTO overexpression (right). Two-way ANOVA was used for comparisons at each time point. **i** The Western blotting assay of FTO in AGS AKATA cells overexpressing wild-type and catalytic mutant FTO. **j** Images (left) and quantification (right) of the cell migration assay of AGS AKATA cells overexpressing wild-type and catalytic mutant FTO. Scale bar: 200 μm. The data in (**b-e**, **h** and **j**) are presented as the means ± SDs. *P* values were calculated by Student’s t test (**b**-**e**, **j**) and two-way ANOVA (**h**). **P* < 0.05, ***P* < 0.01, ****P* < 0.001. GAPDH (**a**) and Vinculin (**f** and **i**) served as loading controls.


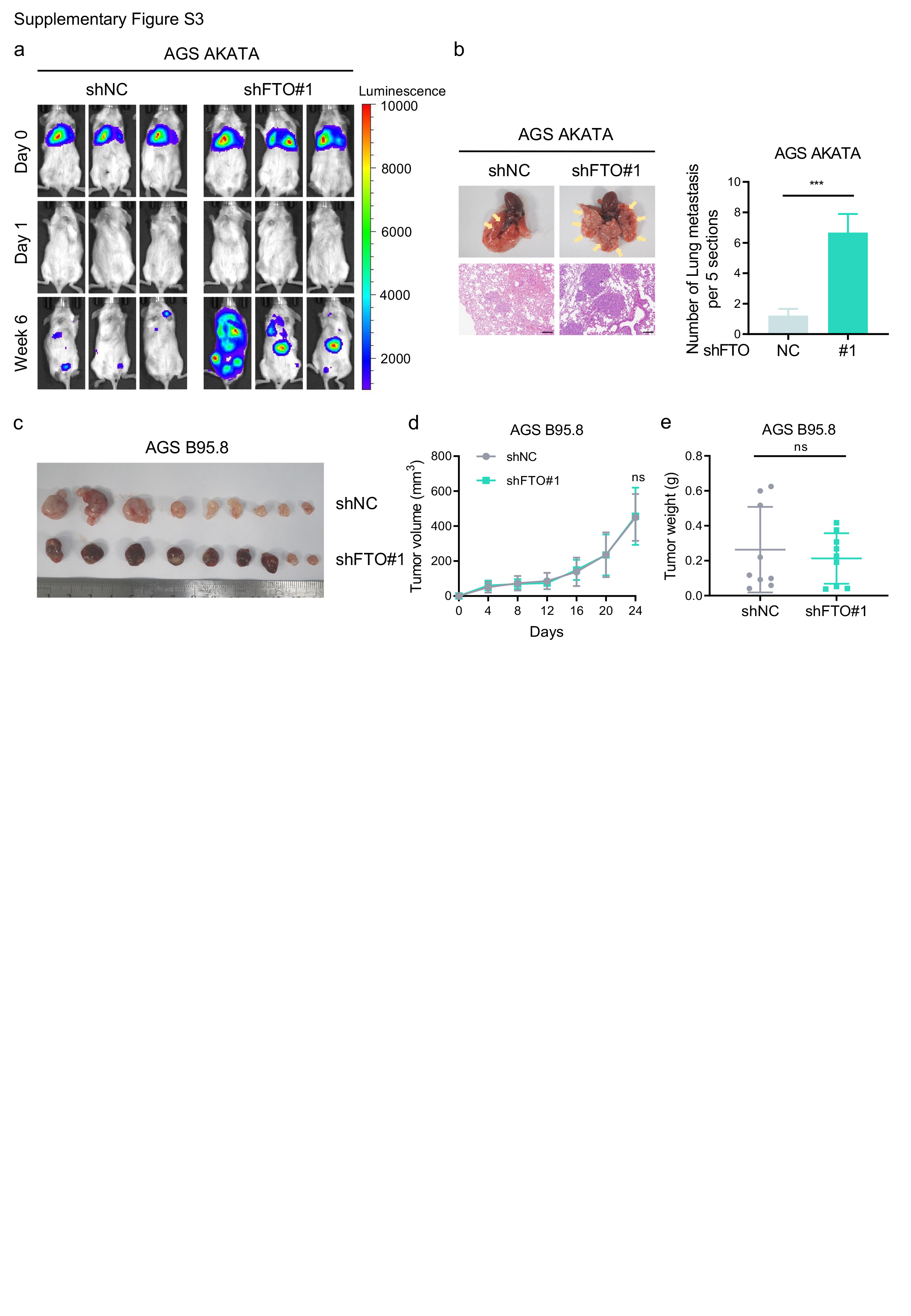


**Supplementary Figure S3.**

**FTO suppressed EBVaGC cell metastasis *in vivo*. a** Bioluminescent imaging of the B-NDG mouse lung metastasis model with shFTO#1 and shNC luciferase-labelled AGS AKATA cells at day 0, day 1 and week 6 (n = 9 per group). **b** Representative specimen and haematoxylin and eosin (H&E) staining photographs (left) of lung metastatic nodules from B-NDG mice injected with FTO knockdown and control AGS AKATA cells via the tail vein, and lung metastatic nodules under a microscope were recorded (right). Scale bar: 200 μm. **c-e** Photograph showing the excised subcutaneous tumours (**c**), tumour volumes (**d**) and weights (**e**) by implanting FTO-knockdown and control AGS B95.8 cells in B-NDG mice (n = 9 per group). Two-way ANOVA and Student’s t test were used for comparisons of tumour volumes (**d**) and tumour weights (**e**), respectively. The data in (**b**, **d** and **e**) are presented as the means ± SDs. *P* values were determined by Student’s t test (**b**, **e**) and two-way ANOVA (**d**). **P* < 0.05, ***P* < 0.01, ****P* < 0.001.


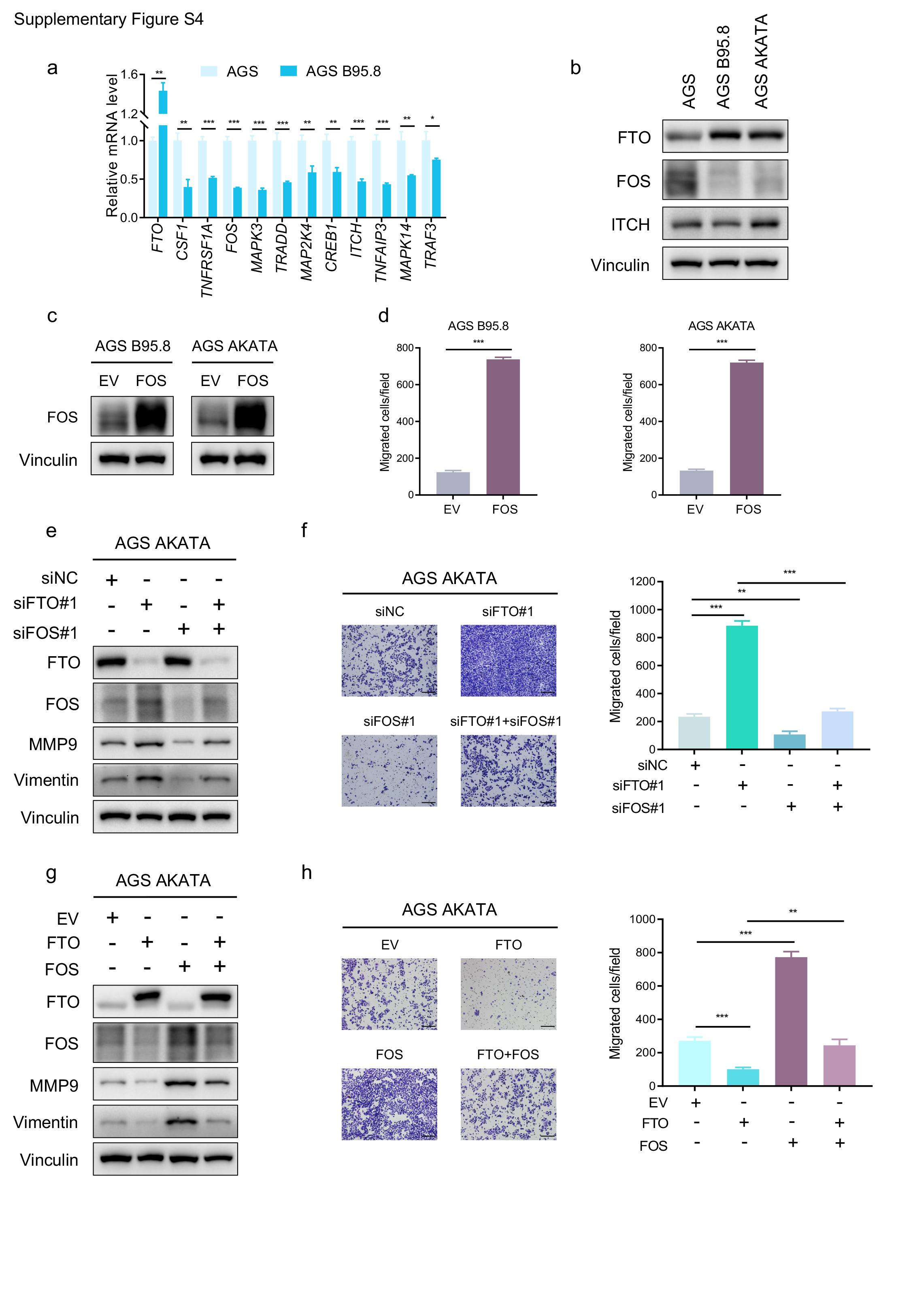


**Supplementary Figure S4.**

**FOS is a functionally critical downstream target of FTO in EBVaGC metastasis. a** RT‒qPCR analysis of mRNA expression of the shared genes in the TNF signalling pathway (CSF1, TNFRSF1A, FOS, MAPK3, TRADD, MAP2K4, CREB1, ITCH, TNFAIP3, MAPK14 and TRAF3) in AGS B95.8 and AGS cells. **b** FOS and ITCH protein levels were detected by western blotting assay in EBVaGC and EBVnGC cells. **c** Immunoblotting of FOS in FOS-overexpressing (FOS) and empty vector (EV) AGS B95.8 (left) and AGS AKATA (right) cells. **d** Quantification of transwell migration assays of AGS B95.8 (left) and AGS AKATA (right) cells upon FOS overexpression and EV. **e** Western blotting of FTO, FOS, MMP9 and Vimentin protein levels in AGS AKATA cells transfected with siNC, siFTO#1, siFOS#1 and siFTO#1 + siFOS#1. **f** Images (left) and quantification (right) of migrated AGS AKATA cells with siNC, siFTO#1, siFOS#1 and siFTO#1 + siFOS#1. **g** Immunoblotting detection of FTO, FOS, MMP9 and Vimentin expression levels in AGS AKATA cells with EV, FTO, FOS and FTO + FOS. **h** Images (left) and quantification (right) of migrated AGS AKATA cells with EV, FTO, FOS and FTO + FOS. Scale bar: 200 μm. The data in (**a**, **d**, **f** and **h**) are presented as the means ± SDs. *P* values were determined by Student’s t test. **P* < 0.05, ***P* < 0.01, ****P* < 0.001. Vinculin was included as a loading control.


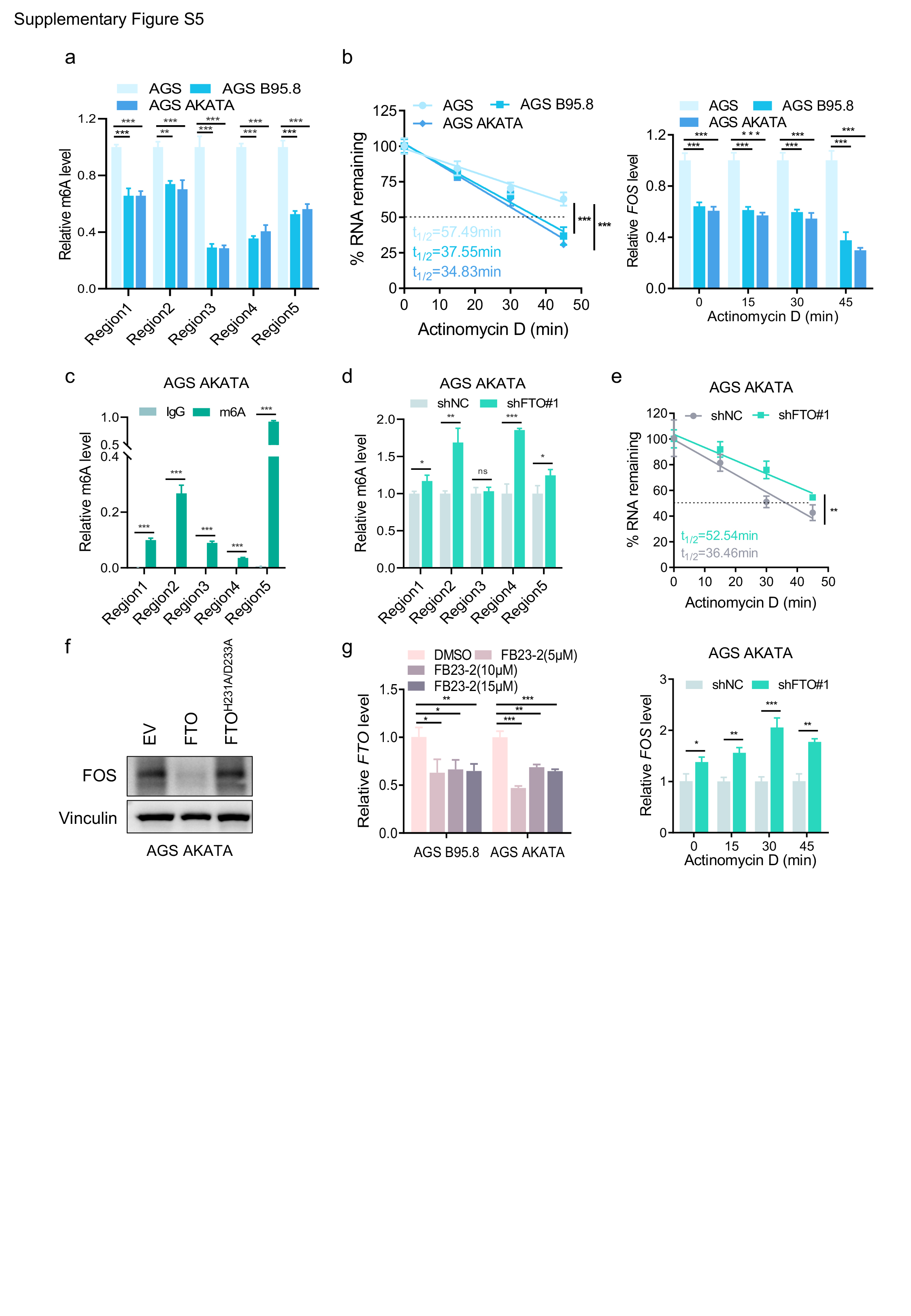


**Supplementary Figure S5.**

**FOS is regulated by FTO-dependent m6A demethylation.** **a** MeRIP-qPCR analysis of m6A enrichment in regions 1-5 of FOS transcripts in EBVnGC and EBVaGC cells. **b** The decay rate (left) and qPCR detection (right) of FOS mRNA at the each time point after actinomycin D (ActD) treatment in EBVnGC and EBVaGC cell lines. **c** MeRIP-qPCR analysis of m6A levels in regions 1-5 of FOS transcripts in AGS AKATA cells. **d** Alterations in m6A enrichment of FOS mRNA regions in AGS AKATA cells after FTO knockdown, as determined by MeRIP-qPCR analysis. **e** The decay rate (top) and qPCR analysis (bottom) of FOS mRNA at indicated times after ActD treatment in FTO-knockdown and control AGS AKATA cells. **f** Western blotting detection of FOS expression in AGS AKATA cells with empty vector (EV), wild-type FTO overexpression (FTO) and H231A/D233A-mutant FTO overexpression (FTO^H231A/D233A^). **g** Relative FTO mRNA expression in EBVaGC cells treated with DMSO, FB23-2 (5 μM), FB23-2 (10 μM) and FB23-2 (15 μM). The data in (**a**-**e**, **g**) are presented as the means ± SDs. *P* values were determined by Student’s t test (**a**, **c**, **d**, **g** and Relative *FOS* level in **b**, **e**) and two-way ANOVA (%RNA remaining in **b, e**). **P* < 0.05, ***P* < 0.01, ****P* < 0.001. IgG was used as the negative control and the relative m6A level was normalized by the input in (**a**, **c** and **d**). Vinculin was included as a loading control.


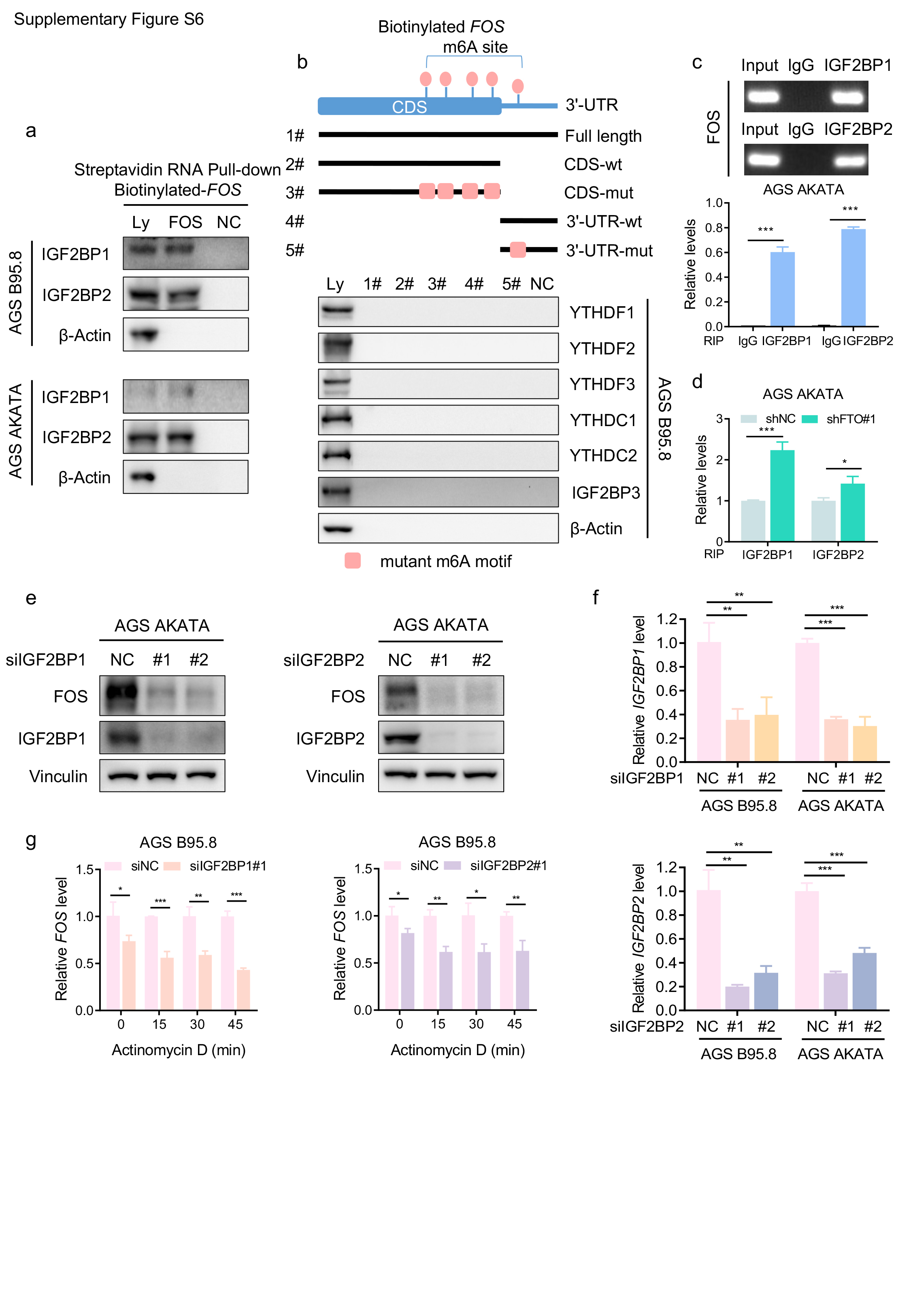


**Supplementary Figure S6.**

**IGF2BP1/2 promotes FOS transcript stability by binding its m6A-modified mRNA. a** Immunoblotting of IGF2BP1/2 with cell lysate (Ly), biotinylated full-length FOS (FOS), and beads only (NC) in EBVaGC cells by RNA pulldown assay. **b** Western blotting of the YTH family and IGF2BP3 with cell lysate (Ly), full-length FOS (#1), the FOS CDS region with or without m6A motif mutation (#2, #3), the FOS 3’-UTR with or without m6A motif mutation (#4, #5) and beads only (NC) in AGS B95.8 cells. **c** Agarose gel electrophoresis (top) and qPCR analysis of RIP assays demonstrated the direct binding between the IGF2BP1/2 protein and FOS mRNA in AGS AKATA cells. **d** RIP assays of the enrichment of IGF2BP1/2 protein binding FOS mRNA in FTO-silenced versus control group. **e** Western blotting of FOS protein levels after IGF2BP1 (left) and IGF2BP2 (right) inhibition in AGS AKATA cells. **f** RT‒qPCR analysis of IGF2BP1 (top) and IGF2BP2 (bottom) mRNA expression levels in EBVaGC cells upon IGF2BP1 (top) and IGF2BP2 (bottom) inhibition. **g** Q-PCR analysis of FOS transcripts at each time point in ActD-treated AGS B95.8 cells after IGF2BP1 knockdown (left) and IGF2BP2 knockdown (right). The data in (**c**, **d**, **f** and **g**) are presented as the means ± SDs. *P* values were determined by Student’s t test. **P* < 0.05, ***P* < 0.01, ****P* < 0.001. β-Actin (**a**, **b**) and Vinculin (**e**) were included as loading controls.


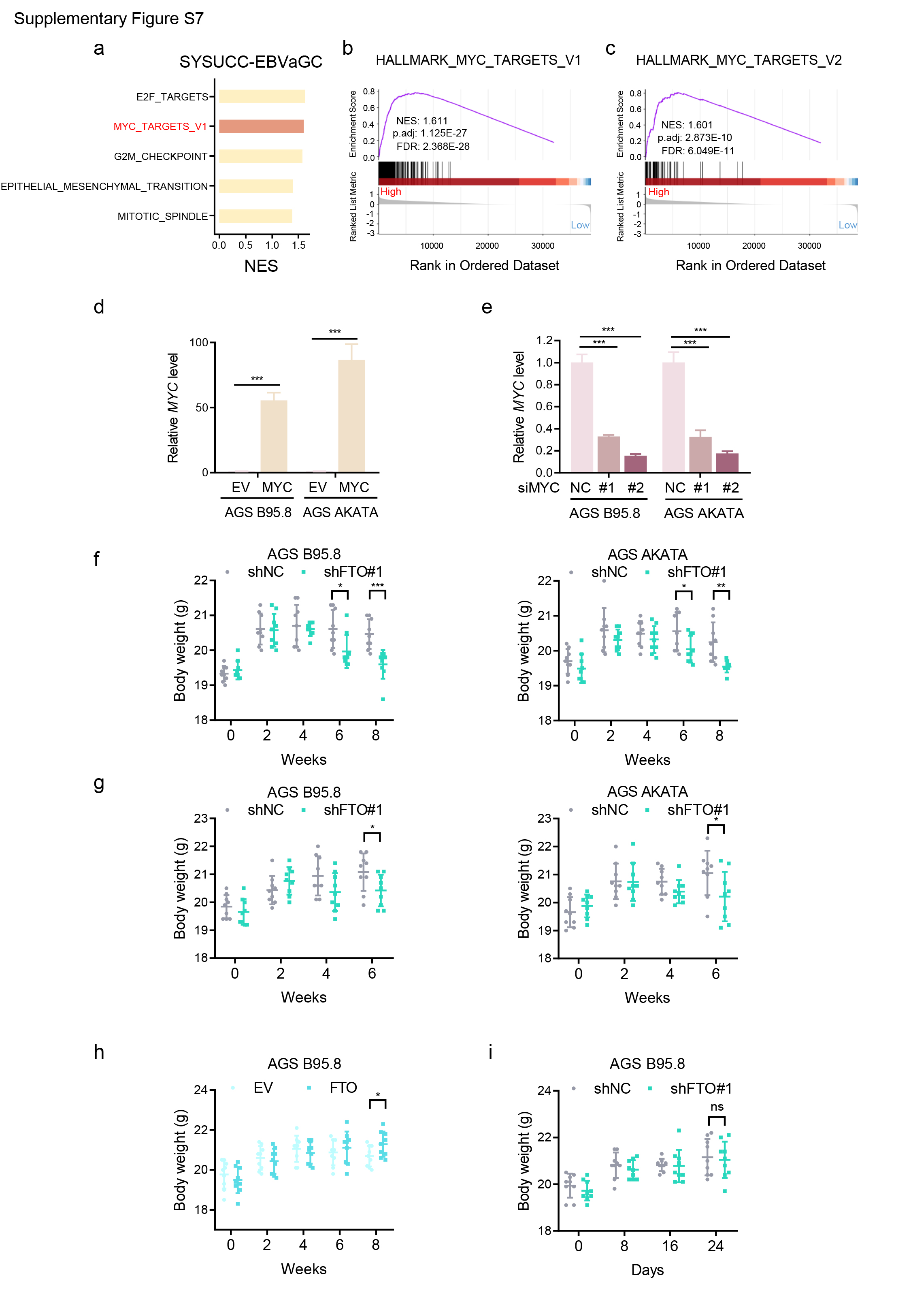


**Supplementary Figure S7.**

**EBV induces FTO expression by the transcription factor MYC in GC. a** Gene set enrichment analysis (GSEA) of differential expression genes in the FTO high expression group versus FTO low expression group. Bar plot illustrating top 5 hallmark pathways sorted by NES. **b, c** GSEA showing that FTO was positively correlated with MYC pathway, and **b** ranks second and **c** ranks eight in the Hallmark enriched pathway set. **d**, **e** RT‒qPCR analysis of MYC mRNA expression in EBVaGC cells upon MYC upregulation (**d**) and MYC downregulation (**e**). **f** the body weight of the B-NDG mice injected intraperitoneally with FTO knockdown and control EBVaGC cells at week 0, 2, 4, 6 and 8. **g** the body weight of the lung metastasis model B-NDG mouse with shFTO#1 and shNC luciferase-labelled EBVaGC cells at week 0, 2, 4 and 6. **h** the body weight data of the lung metastatic model B-NDG mouse in the FTO-overexpressing and empty vector groups at week 0, 2, 4, 6 and 8. **i** the body weight of the B-NDG mice injected subcutaneously with FTO silencing and control AGS B95.8 cells at each time point. The data in (**d**-**i**) are presented as the means ± SDs. *P* values were determined by Student’s t test. **P* < 0.05, ***P* < 0.01, ****P* < 0.001.
